# Functional Divergence and Evolutionary Turnover in Mammalian Phosphoproteomes

**DOI:** 10.1371/journal.pgen.1004062

**Published:** 2014-01-23

**Authors:** Luca Freschi, Mazid Osseni, Christian R. Landry

**Affiliations:** 1 Département de Biologie, Université Laval, Québec, Canada; 2 Institut de Biologie Intégrative et des Systèmes (IBIS), Université Laval, Québec, Canada; 3 PROTEO, The Quebec Research Network on Protein Function, Structure and Engineering, Université Laval, Québec, Canada; National Institute of Genetics, Japan

## Abstract

Protein phosphorylation is a key mechanism to regulate protein functions. However, the contribution of this protein modification to species divergence is still largely unknown. Here, we studied the evolution of mammalian phosphoregulation by comparing the human and mouse phosphoproteomes. We found that 84% of the positions that are phosphorylated in one species or the other are conserved at the residue level. Twenty percent of these conserved sites are phosphorylated in both species. This proportion is 2.5 times more than expected by chance alone, suggesting that purifying selection is preserving phosphoregulation. However, we show that the majority of the sites that are conserved at the residue level are differentially phosphorylated between species. These sites likely result from false-negative identifications due to incomplete experimental coverage, false-positive identifications and non-functional sites. In addition, our results suggest that at least 5% of them are likely to be true differentially phosphorylated sites and may thus contribute to the divergence in phosphorylation networks between mouse and humans and this, despite residue conservation between orthologous proteins. We also showed that evolutionary turnover of phosphosites at adjacent positions (in a distance range of up to 40 amino acids) in human or mouse leads to an over estimation of the divergence in phosphoregulation between these two species. These sites tend to be phosphorylated by the same kinases, supporting the hypothesis that they are functionally redundant. Our results support the hypothesis that the evolutionary turnover of phosphorylation sites contributes to the divergence in phosphorylation profiles while preserving phosphoregulation. Overall, our study provides advanced analyses of mammalian phosphoproteomes and a framework for the study of their contribution to phenotypic evolution.

## Author Summary

Understanding how differences in cellular regulation lead to phenotypic differences between species remains an open challenge in evolutionary genetics. The extensive phosphorylation data currently available allows to compare the human and mouse phosphoproteomes and to measure changes in their phosphoregulation. We found a general conservation of phosphorylation sites between these two species. However, a fraction of sites are conserved at the sequence level (the same amino acid is present in both species) but differ in their phosphorylation status. These sites represent candidate sites that have the potential to explain differences between human and mouse signalling networks that do not depend on the divergence of orthologous residues. Furthermore, we identified several sites where to a phosphorylation site in one species corresponds a non-phosphorylatable residue in the other one. These cases represent clear differences in protein regulation. Recent studies suggest that phosphorylation sites can shift position during evolution, leading to configurations in which pairs of divergent phosphorylation sites are functionally redundant. We identified more than 100 putative such cases, suggesting that divergence in amino acid does not necessarily imply functional divergence when comparing phosphoproteomes. Overall, our study provides new key concepts and data for the study of how regulatory differences may be linked to phenotypic ones at the network level.

## Introduction

Most proteins undergo chemical modifications after their synthesis (post-translational modifications, PTMs). These modifications allow a fine-tuning of protein functions and represent a mechanism to expand the coding capacity of genes [1]. Over the past decade, methods based on mass spectrometry have accelerated the discovery of PTMs [2]–[7]. Each experiment can now detect thousands of modified residues, allowing to probe the functional state of entire proteomes. The PTM that has been studied the most is protein phosphorylation: the addition of a phosphate group to specific amino acids (serine (S), threonine (T) and tyrosine (Y) in eukaryotes). Phosphorylation has been shown to affect protein functions, interactions, stability and localization [8]–[11]. It is thus of fundamental importance to understand how protein phosphorylation evolves within and between species because changes in phosphorylation profiles may cause changes in protein function and regulation and in organismal phenotypes, including disease development (e.g. [12]).

There have been several reports recently on the evolution of phosphoproteomes. For instance, Kim and Hahn [13] identified phosphorylation sites that emerged after the split between humans and chimpanzees and found that these sites are located in proteins involved in crucial biological processes such as cell division and chromatin remodelling. Other studies have looked at the evolution of a subset of phosphoproteomes on a broader evolutionary scale [14], [15]. For example, Boulais and collaborators [14] performed a phosphoproteomics analysis of mouse phagosomal proteins and then compared these proteins to their orthologs from 10 model organisms, from Drosophila to mouse [14]. They observed that the phagosomal phosphoproteome was extensively rewired during evolution, but that some phosphorylation sites have been maintained for more than a billion years, suggesting their importance for phagosomal functions. Finally, other studies looked at the conservation and divergence of entire phosphoproteomes over a broad evolutionary scale [16]–[19] (and reviewed in [20]) in order to understand the evolutionary mechanisms and the constraints acting on phosphorylation sites. These studies found that phosphorylated residues tend to be on average more conserved than their non-phosphorylated counterparts [16], [17] and that this is particularly true for those that were experimentally shown to play functional roles [17].

Most studies that aimed at studying the evolution of phosphoproteomes so far have looked at the evolutionary conservation of phosphorylation sites in several species without knowing if these sites are actually phosphorylated in species other than the reference. In other words, if a phosphorylation site in one species corresponds to a phosphorylatable amino acid in another species, both residues were considered as conserved phosphorylation events. This assumption was necessary because of the lack of phosphorylation data available for more than one species. However, we can hypothesize that residue conservation does not always imply phosphoregulatory conservation. Indeed, sites could be conserved at the residue level but differ in their phosphoregulation due to changes elsewhere in the protein, for instance, the recognition motifs of the protein by kinases and phosphatases [21] or upstream (in *trans*) in the signalling cascade. This aspect has not been addressed by previous studies, except in a few cases [14], [22]. However, identifying such sites is of great interest since sites that differ in their phosphoregulation despite being conserved at the residue level could lead to changes in the architecture of phosphorylation networks and, ultimately, contribute to phenotypic evolution. We examine this issue here.

Another aspect of phosphoproteomes that can be studied using evolutionary analysis is how phosphorylation sites alone or in combination may affect the function of a protein [1]. Many models of phosphorylation site function stress the importance of conformational changes by protein phosphorylation [1], [23], [24]. In other models, phosphorylation sites regulate protein functions without the need for conformational changes but rather through changes in the local charge of the protein [25], i.e. simply through bulk electrostatics. A corollary of this last model is that the protein phosphorylation code is redundant, *i.e.* that phosphorylation sites can change their position over time and still maintain their biological function as long as the number of sites in a given protein region is preserved, without affecting organismal phenotypes. By looking at the patterns of evolution of phosphorylation sites, one could find traces of this redundancy by studying rapid phosphorylation site evolutionary turnover (phosphorylation site gains and losses). This evolutionary turnover has been invoked for interpreting the global rapid pattern of evolution in different species [15]–[17], [26]–[28]. However, evidence for positional redundancy of phosphorylation sites is relatively limited. Two independent pieces of evidence come from the cell cycle phosphorylation networks. Moses and collaborators [29] studied the evolution of cyclin-dependent kinase (CDK) consensus phosphorylation sites of the yeast pre-replicative complex [30]. They found that although orthologous proteins contained clusters of CDK consensus sites, the position and the number of phosphorylatable sites were not conserved, suggesting that phosphorylation sites tend to shift their positions during evolution. In a more recent investigation, Holt and collaborators [31] compared the positions of 547 phosphorylation sites on 308 Cdk1 substrates *in vivo* in the budding yeast and their orthologous sites in other fungi. They found that the precise positioning is conserved only in the very closely related species. However, in both cases the phosphorylation status of the sites in other species was not investigated so it is not clear whether the phosphorylation sites were absent from the orthologous proteins or if they actually shifted during evolution through gains or losses to another position. The extent to which phosphorylation site positional redundancy plays a role in overall phosphoproteome turnover therefore awaits comprehensive phosphorylation data from closely related species, which we have assembled here.

We performed an integrated analysis of phosphorylation site evolution between the human and mouse proteomes using a large dataset of phosphorylation sites [6], [32]–[37]. These two phosphoproteomes are the ones for which we have the greatest amount of phosphoproteomics data between closely related species. We estimated the extent of divergence and conservation between the two phosphoproteomes and we investigated whether phosphorylation site evolutionary turnover could contribute to this divergence.

## Results and Discussion

### Conservation and divergence between human and mouse phosphoproteomes

**Figure 1:**
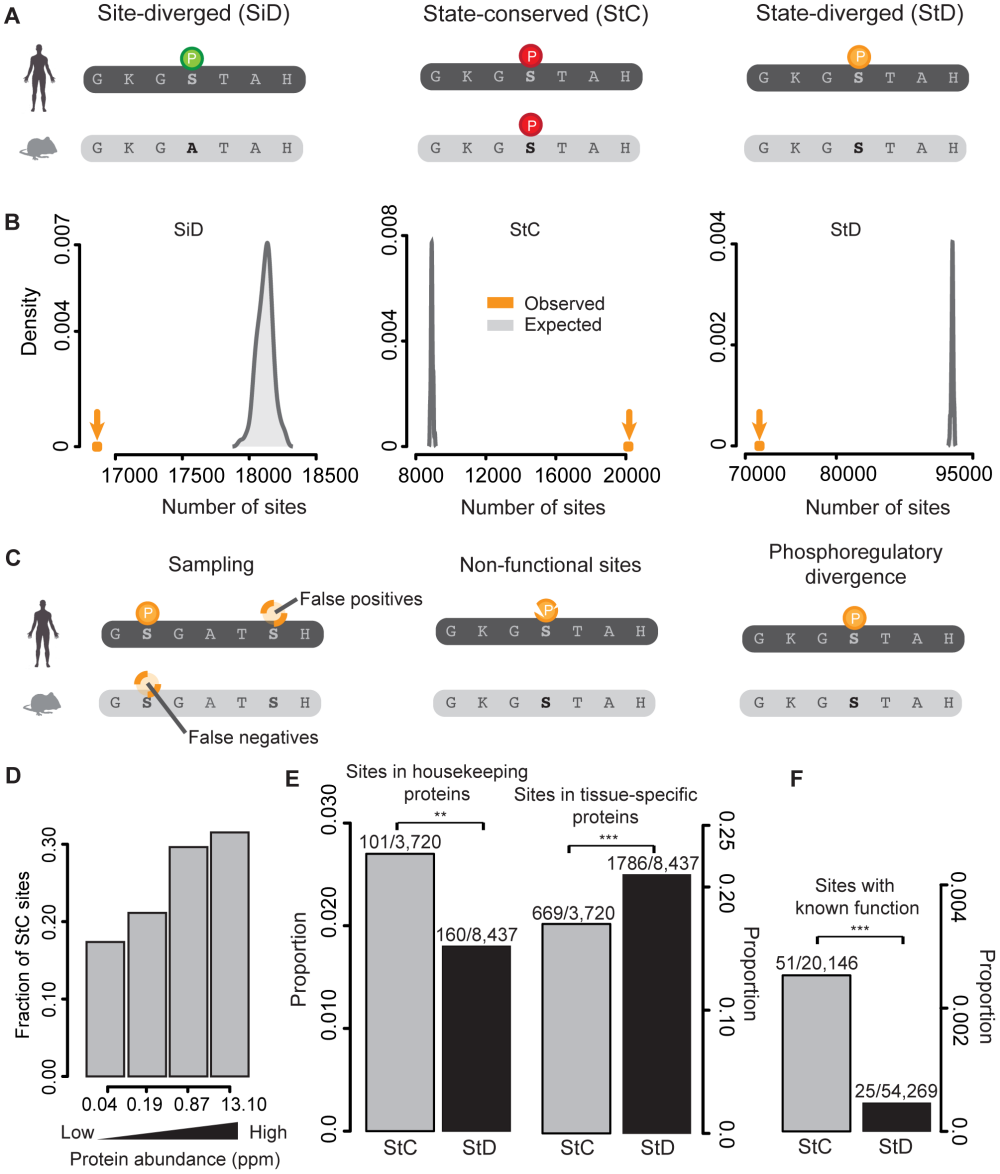
Purifying selection is acting on mammalian phosphorylation sites and their phosphorylation status. (A) Site-diverged (SiD) sites are orthologous residues where one is phosphorylated and the other is a non-phosphorylatable amino acid (any amino acid but S, T and Y). State-conserved (StC) sites are orthologous phoshorylatable residues (S, T, Y) that are both reported to be phosphorylated. Finally, state-diverged (StD) sites are orthologous phosphorylatable residues for which only one of the two is phosphorylated. Circles with the P symbol indicate residue phosphorylation. Colors indicate the different categories of sites. (B) Number of observed SiD, StC and StD sites and their respective expected distributions as estimated by randomizing mouse phosphorylation sites. (C) Three scenarios for StD sites: false positive and false negative identifications; rapidly evolving non-functional phosphorylation sites; divergence in phosphoregulation. (D) Relationship between state-conservation and protein abundance. The four classes of protein abundance have the same number of proteins. (E) Comparison of the proportion of StC and StD sites in housekeeping and tissue-specific proteins. (F) Comparison of the proportion of sites with known functions present in StC and StD sites.

We assembled a dataset of human (n = 106,877) and mouse (n = 54,400) phosphorylation sites by collecting data from 7 different databases and experimental studies [6], [32]–[37] (Table S1). We successfully mapped 128,705 sites onto 11,150 human and mouse orthologous proteins: 86,065 in humans and 42,640 in mouse (Figure S1). As previously observed [17], [38], phosphorylation sites are preferentially located in disordered regions of proteins (observed vs. expected proportions: 0.69 vs. 0.62, p-value = 2.2×10^−16^). Given this asymmetry in the localization of phosphorylation sites, we generated all the null models of our analyses by respecting the proportion of sites in these two structural categories. Our dataset allows comparing the human and mouse phosphoproteomes using both sequence information and the phosphorylation status of each site. Accordingly, we classified orthologous sites into three classes following Freschi *et al.* [28] (Figure 1A): i) Site-diverged (SiD): sites phosphorylated in one species and non-phosphorylatable in the other; ii) State-conserved (StC): sites phosphorylated in both species; iii) State-diverged (StD): sites that are conserved at the residue level but that have been reported to be phosphorylated in only one of the two species.

In order to examine the extent of conservation of phosphorylation between human and mouse, we estimated the fraction of sites belonging to each of these three categories compared to the total number of sites that are phosphorylated in human, mouse or in both species. We first looked at phosphorylation site divergence. We found that 16,863 sites (16% of the sites that are phosphorylated in human or mouse or both species) are SiD (Figure 1B). These sites are about 1% less abundant than random expectations obtained by shuffling the phosphorylation statuses of S/T/Y residues (Figure 1B), suggesting that purifying selection is acting on phosphorylation sites to maintain their function but to a limited extent, as previously observed with different approaches (e.g. [17]). These sites, if functional, are expected to reflect differences in phosphoregulation between human and mouse. However, a fraction of these SiD sites might be positionally redundant site pairs such that the functional divergence may be overestimated (see below).

We examined other types of conservation and divergence. We first found that 20,146 phosphorylation sites (18% of the sites that are phosphorylated in human or mouse or both species, Figure 1B) are StC. This proportion is 2.5 times greater than what is expected by chance alone (Figure 1B). We observed this strong signal for conservation in both disordered and ordered regions (Figure S2). These results suggest an overall conservation of the phosphorylation profiles between the two species, most likely as a result of purifying selection acting to maintain the phosphoregulation of these sites. We performed a similar analysis on clusters of poly-S/T/Y (stretches of two or more consecutive S/T/Y residues) rather than single residues and found the same patterns of conservation and divergence (Figure S3).

Despite an overall signal of conservation on the phosphorylation status of proteins, the most represented category of sites in our dataset is StD sites (71,550 sites or 66% of the sites that are phosphorylated in human, mouse or both species). Three different non-exclusive scenarios could explain this large number of StD sites (Figure 1C). The first one implies that state divergence results from an incomplete coverage of phosphoproteomic data, which means that the phosphoproteomes of the two species might have been undersampled, for instance sampled at different depths or in different conditions or tissues (e.g. [6]). The second scenario is that a large fraction of the StD sites identified might result from non-functional phosphorylation sites. Non-functional phosphorylation sites evolve rapidly [17] and could therefore lead to the poor conservation on the phosphorylation status we observed. The third scenario is that a fraction of StD phosphorylation sites is actually diverging in its regulation. Finally, state-divergence could also be inflated by false positive identifications in one species or the other.

**Figure 2:**
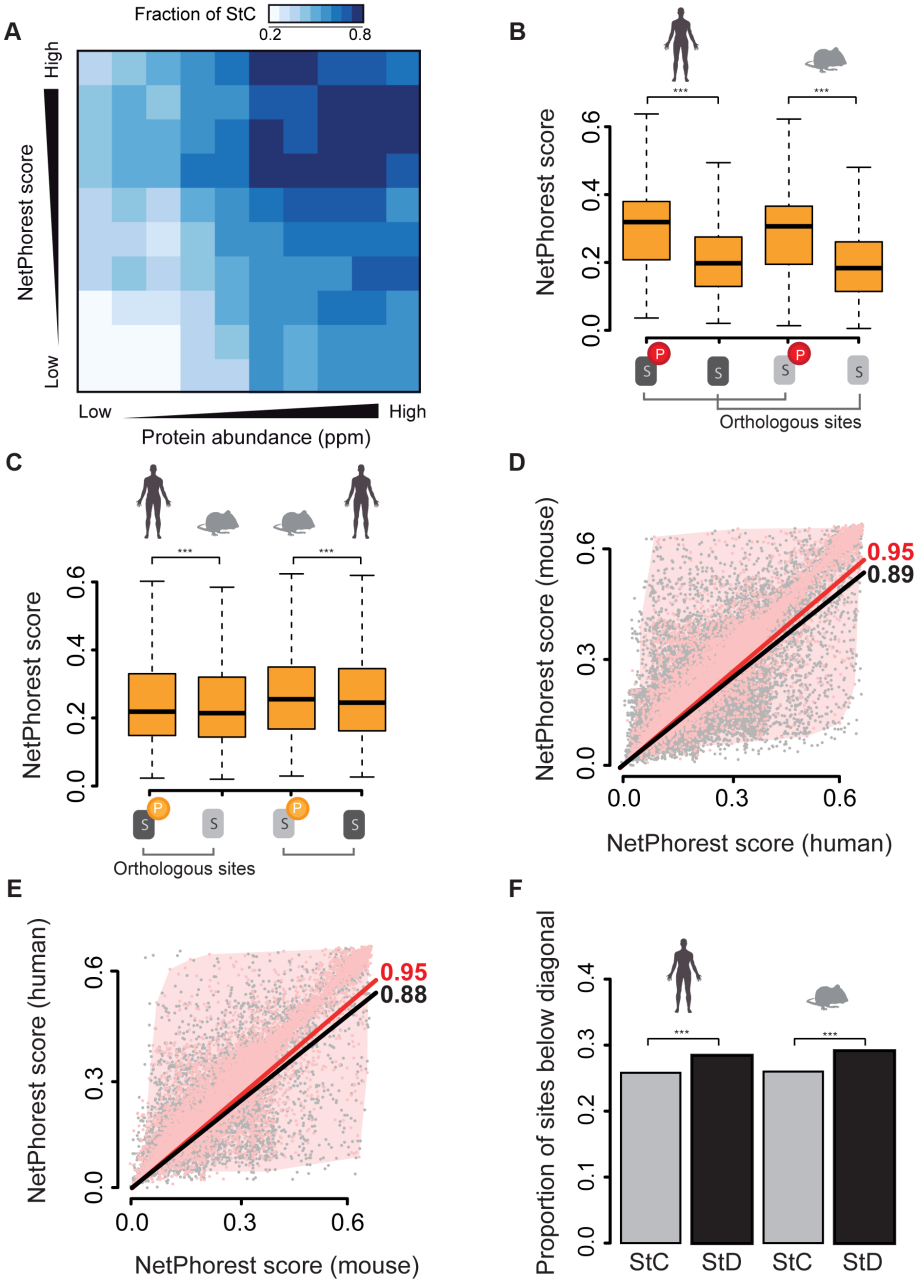
Analysis of NetPhorest scores for the different classes of sites. (A) Fraction of StC sites as a function of NetPhorest scores and protein abundance. (B) Comparison of NetPhorest scores for human and mouse phosphorylated and non-phosphorylated residues (Wilcoxon tests). (C) Comparison of NetPhorest scores for StD sites (Wilcoxon tests). (D) Correlation between human and mouse NetPhorest scores for StC sites (black) and StD sites phosphorylated in human but not in mouse (red). (E) Correlation between human and mouse NetPhorest scores for StC sites (black) and StD sites phosphorylated in mouse but not in human (red). (F) Proportion of phosphorylated sites that have higher NetPhorest scores compared to their corresponding site in the other species for StC and StD sites. Comparisons of human and mouse scores calculated with position weight matrices are shown in Figure S4. *: p-value<0.05; **: p-value<0.01; ***: p-value<0.001.

We examined which scenario or scenarios were compatible with our data. According to the first scenario, StD may mostly result from false-negative phosphorylation sites in the data. This is certainly the case for an important part of the data as our dataset contains twice as much phosphorylation data for humans than mouse, and humans are not expected to have more phosphorylation sites than mouse. We reasoned that if state-divergence is caused by false-negatives in the datasets, we would expect to see the fraction of StC to increase as a function of protein abundance, since highly abundant proteins are more likely to be sampled in both species than rare proteins. Indeed, we found that the proportion of state conserved sites almost doubles between the two extreme classes of abundance (Figure 1D, see also Figure 2A). Admittedly, this effect could also be caused by the fact that phosphoregulation is more conserved on highly-expressed proteins but it is unlikely, as it was recently shown that abundant proteins are enriched in non-functional phosphorylation sites [20] that evolve relatively rapidly [17]. In addition, only conserved residues are considered in this analysis.

We also examined whether StC or StD phosphorylation sites were more likely to be found in housekeeping or tissue-specific proteins. Housekeeping proteins are expressed in all tissues, while tissue-specific ones are expressed in one or a few tissues. Accordingly, if StD sites are affected by false negatives we would expect to find them preferentially in tissue-specific proteins. We examined the dataset of housekeeping genes [39] and tissue-specific genes [40] and found that StC sites are preferentially found in housekeeping proteins compared to StD sites (proportions: 0.027 vs. 0.019, p-value = 0.005, Figure 1E), while the trend is reversed if we look at tissue specific proteins (proportions: 0.268 for state diverged vs. 0.219 for StC, p-value = 6.1×10^−5^, Figure 1E). This result is in agreement with our hypothesis that StD sites are affected by false negatives, although this effect could be due to the fact that phosphoregulation is more conserved on housekeeping proteins.

In order to examine whether non-functional phosphorylation sites could contribute to poor state-conservation between species, we used a manually curated dataset of functional phosphorylation sites compiled by Landry and collaborators [17]. Functional sites were identified as sites for which a phenotype was observed when phosphorylatable residues were mutated. If non-functional sites contribute to state-divergence, we would expect functional sites to be overrepresented in StC sites. We found that StC sites are enriched in functional phosphorylation sites compared to StD sites (proportions: 0.0025 vs. 0.00046, p-value<1.19×10^−14^, Figure 1F). This observation suggests that a fraction of the StD sites we identified might be non-functional phosphorylation sites, which would explain their poor conservation status between species. It is important to consider that in both cases these observations are not biased by residue conservation as both StC and StD categories are composed of only phosphorylatable residues.

### A role for state-diverged sites in phosphoproteome divergence

Our observation that the majority of StD sites might result from false-negative phosphorylation site identifications or might be non-functional does not rule out the possibility that at least some of these sites could be actual StD sites that diverge in regulation, for instance due to the sequences surrounding the phosphorylated residues. Kinase recognition motifs on substrates are difficult to compare directly due to their degeneracy [21]. We therefore relied on kinase prediction tools for our analyses. We assigned each site to a protein kinase using the NetPhorest classifier [41] to associate protein kinases with all phosphorylation sites based on the site flanking sequences. NetPhorest classification is based on an atlas of consensus sequence motifs that covers 179 kinases and 104 phosphorylation-dependent binding domains and was built using *in vivo* and *in vitro* experimental data [41]. If a site is phosphorylated in one species but not in the other, the sequences surrounding the phosphorylatable residue should match a kinase consensus motif better for the phosphorylated site than for the orthologous non-phosphorylated one. Given that NetPhorest provides a score (from 0 to 1) for many possible kinase-substrate associations, we selected the kinase having the best NetPhorest score and we used this score as a proxy to assess the probability of a given site to be phosphorylated. We relaxed this assumption in some of our analyses. In addition, we performed the same analyses directly using a collection of position weight matrices derived from mammalian kinases and the results are in agreement with what we find with the NetPhorest predictions (Figure S4).

We first examined whether there was an association between S/T/Y phosphorylation and NetPhorest scores and found that the probability for a site to be phosphorylated strongly increases with increasing NetPhorest scores in both mouse and human data (Figure S5). Another result in support of this observation is that the fraction of state conserved sites increases as a function of NetPhorest scores (Figure 2A) and this relationship is independent from protein abundance. We also found that prediction scores are very similar for StC sites (median scores: 0.32 for the human phosphorylation sites *vs*. 0.32 in mouse ones, p-value = 0.54) and higher than those of sites conserved at residue level but non-phosphorylated in both species (median scores: 0.32 for StC *vs.* 0.20 for non-phosphorylated residues, p-value = 2.2×10^−16^; Figure 2B and Figure S6A–B). This confirms again a strong association between NetPhorest scores and the probability that a site is phosphorylated. Surprisingly, we found that scores of StC sites were also higher than the scores of the phosphorylated residues in the StD class (median scores: 0.32 *vs.* 0.22 for humans, p-value = 2.2×10^−16^; 0.32 vs. 0.26 for mouse, p-value = 2.2×10^−16^; Figure 2B–C and Figure S6A–B). This means that sites that are conserved and phosphorylated in both species have a significantly better match to consensus kinase motifs than those that are conserved at the residue level but phosphorylated in one species only.

There are several possible explanations for these differences. First, this result could derive from how predictive tools have been developed. For instance, phosphorylation sites may be more often studied on abundant proteins, which would imply that kinase prediction tools are better trained at recognizing phosphorylation sites present on abundant proteins. We tested this hypothesis and found that there is no increase in the average NetPhorest scores as a function of protein abundance (Figure S7), showing that the NetPhorest classification is not biased towards sites present in highly abundant proteins. Another possibility is that StD sites contain a significantly higher proportion of false-positive phosphorylation sites compared to StC sites, as the latter have been found to be phosphorylated in the two species in completely independent experiments and thus have much stronger experimental support. Indeed, false positive sites would have low NetPhorest scores, similar to non-phosphorylated ones and would therefore contribute lowering the average NetPhorest score for the residues that are phosphorylated in StD sites compared to StC sites. A third possibility is that StD sites could contain a proportion of non-functional phosphorylation sites with non-consensus motifs as shown before by Landry and collaborators [17] who found that phosphorylation sites matching kinase motifs have a higher degree of evolutionary conservation and are thus more likely to be functional. Altogether, these results suggest that the match to a consensus sequence motif could be used to the prioritization of phosphorylate sites for downstream functional analysis in phosphoproteomics experiments.

**Figure 3:**
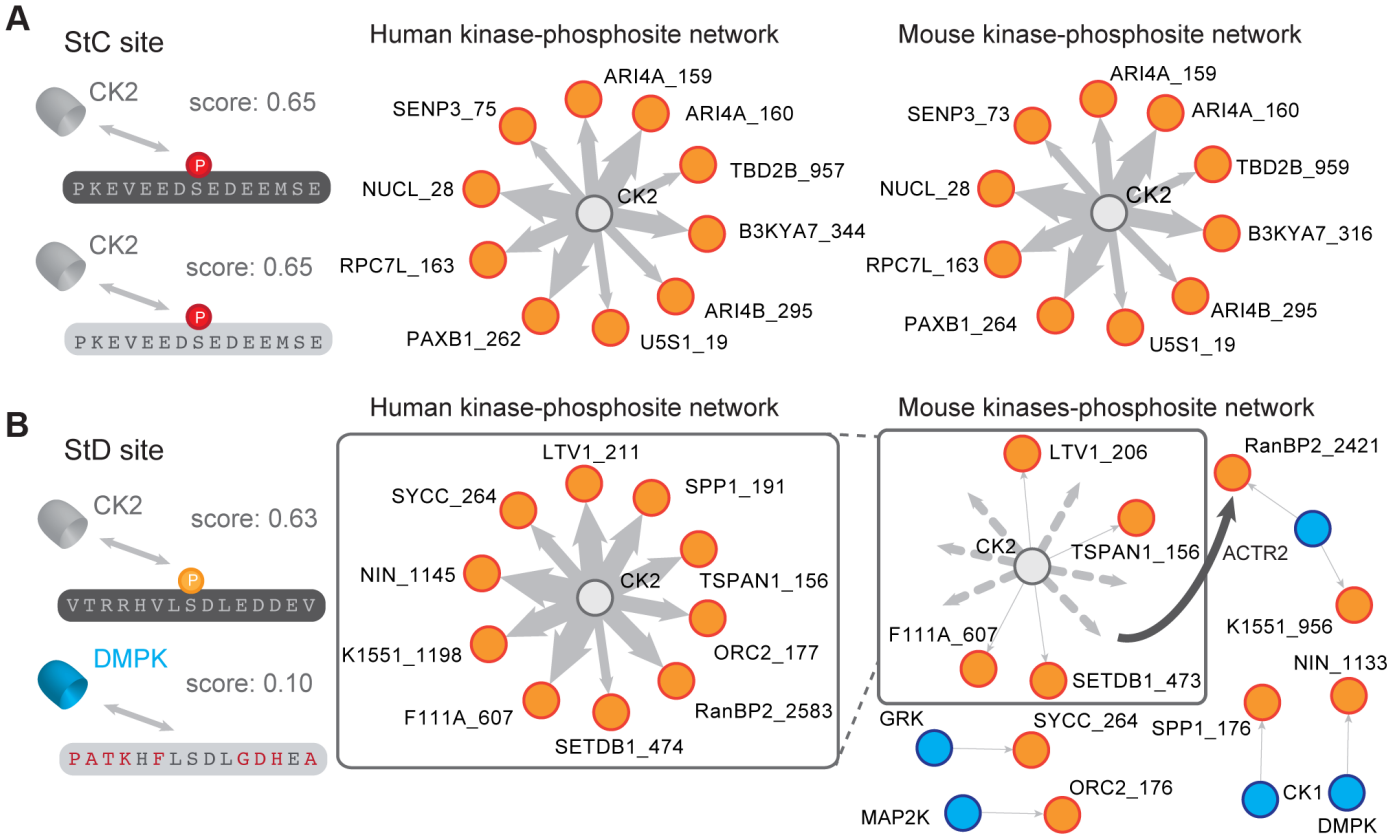
Comparison of a pair of StC and StD sites. (A) Example of StC site (human protein: NUCL; site S28). Both sites are predicted to be phosphorylated by the same kinase (CK2) by NetPhorest. The human and mouse kinase-phosphorylation networks are shown for the 10 StC sites with the highest NetPhorest scores (Table S2). The width of the edges is proportional to the NetPhorest score. (B) Example of StD site (human protein: NIN; site S1145). The two phosphorylation sites are predicted to be phosphorylated by different kinases (human: CK2, mouse DMPK) by NetPhorest. The human and mouse kinase-phosphorylation networks are shown for the 10 StD sites with the highest difference in NetPhorest scores (Table S2). Dotted lines represent predicted kinase-phosphorylation site associations that have been rewired in mouse considering the human network as reference.

Despite these potentially confounding factors, we found evidence that StD is at least partly caused by divergence in regulatory motifs. We found that scores of phosphorylated StD sites are significantly higher than those of their non-phosphorylated orthologous counterparts in both pairwise comparisons (phosphorylated in human *vs.* non-phosphorylated in mouse, median scores: 0.216 vs. 0.214, p-value = 3.93×10^−5^; phosphorylated in mouse vs. non-phosphorylated in humans, median scores: 0.255 vs. 0.245, p-value = 6.38×10^−5^; Figure 2C). The fact that we see the effects in both directions rules out the possibility that NetPhorest scores are systematically higher in humans. In order to identify among the set of StD sites the ones that have the potential to be true StD sites, we directly compared matching orthologous NetPhorest scores of StC and StD sites. We found a strong correlation between the NetPhorest scores for StC sites (rho = 0.95, p-value<2.2×10^−16^) and a weaker correlation between the scores of the StD sites, and this both for those phosphorylated in humans but not in mouse (rho = 0.89, p-value<2.2×10^−16^, Figure 2D) and for those phosphorylated in mouse but not in humans (rho = 0.88, p-value<2.2×10^−16^, Figure 2E). This result is confirmed when comparing the proportion of StD sites having higher scores in humans than in mouse to the same proportion calculated for StC. We found a slight but significant excess of StD sites having higher scores in human than in mouse compared to StC sites (proportions: 0.284 vs. 0.258, p-value = 8.69×10^−13^, Figure 2F). We found similar results for the StD sites having higher scores in mouse compared to humans (proportions: 0.291 vs. 0.261, p-value = 8.69×10^−11^, Figure 2F). By summing up all these excess StD sites that show high NetPhorest scores in one organism but low scores in the other we concluded that that at least 5% of the StD sites (either phosphorylated in human or mouse) present in our dataset have the potential to be sites that are differentially regulated between species, despite a conservation of the actual phosphorylatable residues. Our results do not depend on the NetPhorest algorithm as we performed the same analyses using position weight matrices available from the literature [42]–[53] and all of our conclusions about StC and StD sites were mirrored in these tests, as shown in Figure S4. Overall, our results show that in addition to the actual divergence in phosphorylated sites (SiD), a significant fraction of the mouse and human phosphoproteomes have diverged through changes in the kinase recognition motifs. These changes in the phosphoregulatory status of proteins represent changes in the protein regulatory network, as illustrated for a particular subnetwork in Figure 3. Potential StD sites are located in proteins that have fundamental cellular functions, making them good candidates for the investigation of species-specific mechanisms of regulation. Further examples are available in Table S2.

### Evolutionary turnover of mammalian phosphorylation sites

We next examined whether the positional turnover of phosphorylation sites could contribute to SiD between mouse and humans. One prediction of this model is that sites that are lost in one lineage could be compensated for by the gain of other sites in the proximity [28]. Similarly, sites could change their positions as a result of insertions and deletions in the surrounding regions. In order to test this prediction, we developed an algorithm to identify evolutionary clustered sites [28], *i.e.* pairs of sites that are SiD between mouse and humans and that are closer to each other in the linear protein space than expected by chance alone (Figure S8).

We found that 123 site pairs belonging to 68 proteins show significant evolutionary clustering of SiD phosphorylation sites (Table S3; alignments are available in Dataset S1). Ninety percent of the proteins that contain evolutionary clustered site pairs have only one or two of them (Figure S9) with few exceptions (Table S4). This number also excludes proteins for which we found a high number of evolutionary clustered site pairs due to large clusters of sites that we did not consider (NOL8, 10; KI67, 27; MDC1, 180 site pairs). The median NetPhorest score for these sites is 0.29, suggesting that they are likely to be phosphorylated and not false-positives (0.20 is the median score for non-phosphorylated residues while 0.32 is the median score for phosphorylated residues). The typical window within which we found significant clustering between SiD sites is 10 amino acids (Figure S10) and approximately 80% of the sites are less than 40 amino acids distant in the alignment. The observed number of site pairs (n = 123) is likely an underestimate of the contribution of evolutionary site turnover because we need many possible configurations in the neutral model to identify them and phosphorproteomes have likely been under sampled. We found that the proportion of proteins that show significant evolutionary clustering increases with the proportion of available sites (Figure S11). Furthermore, we found that the number of evolutionary clustered sites is correlated with protein size (rho = 0.26, p-value = 0.03) and may thus be biased towards large proteins.

**Figure 4:**
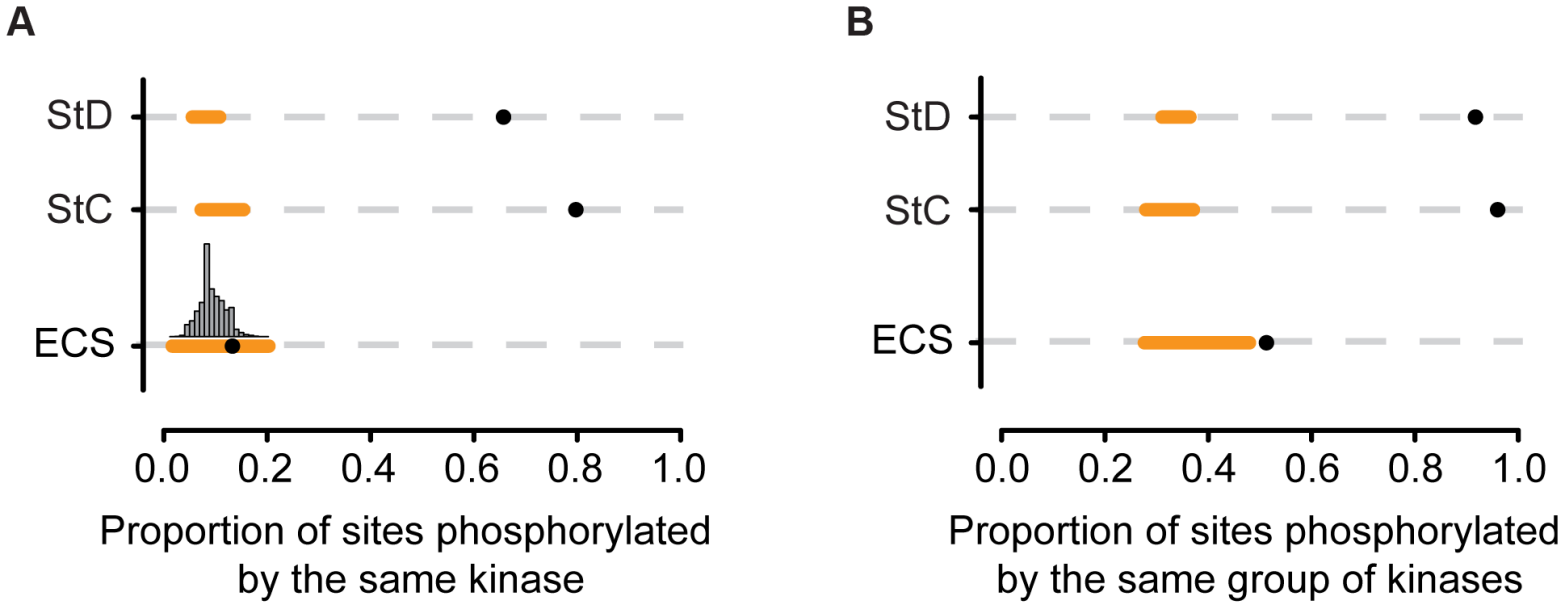
Proportion of sites that are phosphorylated by the same protein kinase. (A) Proportion of sites phosphorylated by the same kinases (NetPhorest predictions) for the different categories of sites (StD: state diverged, StC: state conserved, ECS: evolutionary clustered sites). Black dots represent the observed proportion. Orange lines represent the range of proportions expected by chance alone. P-values for StC and StD: <0.0001; p-value for ECS: 0.03. The histogram shows the distribution expected proportions for ECS. A similar analysis was performed using position weight matrices (Figure S4). (B) Proportion of sites phosphorylated by one or more shared kinases (kinase group) among the three best kinases predicted to be associated with each site according to NetPhorest. P-values for StC, StD and ECS: <0.01.

If these clustered SiD sites were functionally equivalent at the network level between the two species, we would expect them to be phosphorylated by the same kinases or group of kinases. We used again NetPhorest to test this hypothesis. We determined the proportion of StC, StD and evolutionary clustered sites that were likely to be phosphorylated by the same kinases or group of kinases (overlap of one or more kinases among the three best kinases predicted by NetPhorest) [19] and we compared these observations to the random expectations obtained by shuffling the mouse kinase-substrate associations. We found that the proportion of StC and StD sites predicted to be phosphorylated by the same kinases or group of kinases was more than 7 times greater than expected by chance alone, suggesting that, globally, these sites tend to be phosphorylated by the same kinases or group of kinases (Figure 4A–B). We found a slightly significant tendency (p-value = 0.03) for the evolutionary clustered sites to be phosphorylated by the same kinase (Figure 4A). We then performed the same analysis, but considering the three best kinases found by NetPhorest assuming that phosphorylation sites could be functionally conserved if they are phosphorylated by closely related kinases as well, as in Tan *et al.* [19]. We found that evolutionary clustered sites were 1.4 times more likely to be phosphorylated by the same group of kinases than expected by chance alone (p-value<0.01; Figure 4B). This result suggests that, in general, many evolutionary clustered sites may actually be functionally equivalent. Finally, we performed this analysis using position weight matrices available from the literature [42]–[53] and found qualitatively similar results (Figure S4F).

**Figure 5:**
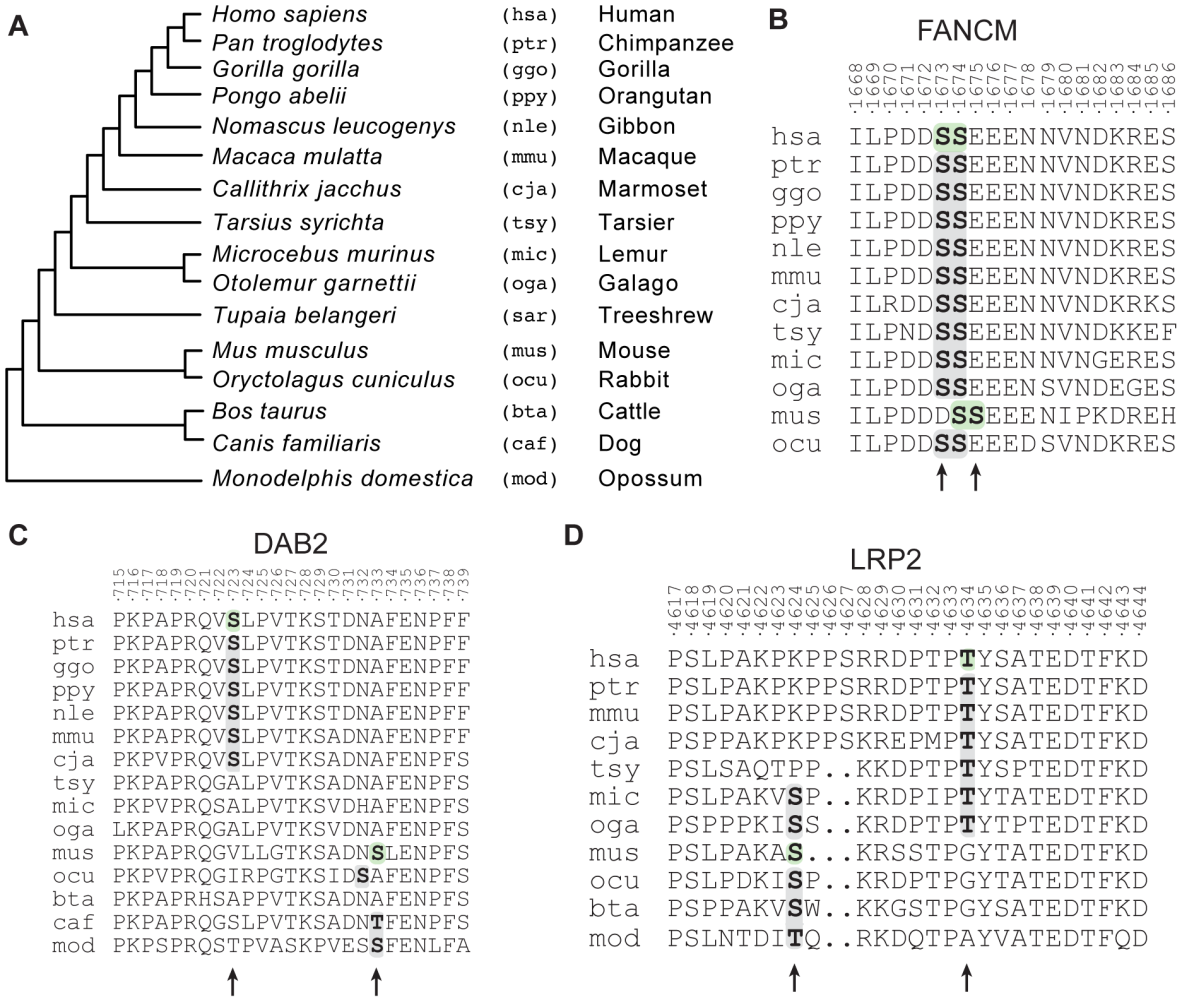
Evolutionary histories of candidate functionally redundant site pairs. (A) Phylogeny of the species considered for the analysis of evolutionary clustered sites. For all species we show the species name, the three-letter identifier and the common name. (B) Alignment of the Fanconi anemia group M protein (FANCM). Evolutionary clustered sites are indicated in bold. Residues that have been reported to be phosphorylated are on a green background. (C) Alignment of the disabled homolog 2 protein (DAB2). (D) Alignment of the low-density lipoprotein receptor-related protein (LRP2).

Evolutionary clustered sites could arise through losses and gains of phosphorylation sites in the two lineages. Our algorithm identifies evolutionary clustered sites, but it cannot tell whether these represent gains of phosphorylation sites that compensated for deleterious losses in the same lineage or whether they were simply the result of indels that affected the position of the sites in the human and mouse protein alignments. We therefore aligned the mouse and human proteins with several orthologs belonging to species that diverged after the human-mouse divergence (Figure 5A) and manually curated the data in order to identify the possible evolutionary steps that led to these configurations of phosphorylation sites.

We manually identified many cases (n = 17, 14%) of evolutionary clustered sites that were most likely caused by indels changing protein length and thus alignment. An example is in the Fanconi anemia group M protein, an ATPase implicated in DNA repair [54] in which S1673 and S1674 are shifted towards the C-terminal in the mouse lineage (Figure 5B). The remaining 86% (n = 106) of the cases of evolutionarily clustered sites could not be simply explained by indels and may thus represent compensatory evolutionary events. We observed such a case in the protein DAB2 (human site: S723; mouse site: S731), which plays a potential role in ovarian carcinogenesis [55] (Figure 5C). The human S723 has been gained after the split of the Haplorrhini from the other primates, while the second one (S731) has been lost after the split between the rodents and the primates. Another example involves the human T4634 and the mouse site S4632 on LRP2 (Figure 5D). This protein is a membrane receptor of absorptive epithelial cells. Mutations in this protein are associated with Donnai-Barrow syndrome, a genetic syndrome that leads to defects in vision, hearing, craniofacial features and structural abnormalities in brain [56]. In this case the human T4634 site appeared in primates after the split from rodents, while the mouse S4632 site was lost after the split of the Strepsirrhini from the other primates. The biological function of these phosphorylation sites has not been determined but they represent prime candidates for exploring, at the molecular level, the positional redundancy of phosphorylation sites.

Here we compared the human and mouse phosphoproteomes in order to gain a detailed picture of phosphoregulatory conservation and divergence between these two species. We found that, globally, phosphorylation sites tend to be conserved between human and mouse. By using phosphorylation data from both species, we showed that the number of the sites that are phosphorylated in both human or mouse is 2.5 times higher than expected by chance alone. In addition, we estimated phosphorylation status divergence. We found that the majority of phosphorylation sites that are conserved at the residue level between human and mouse are actually divergent with respect of their phosphorylation status (StD sites). While this is most likely largely due to incomplete coverage between the two species, we showed that at least 5% of the StD sites are actually diverging at the kinase-substrate interaction level. We also found that phosphorylation sites that are phosphorylated in both species are more likely to be functional and have higher kinase assignment scores, suggesting that this conservation criterion could be used to prioritize phosphorylation sites for further characterization [17], [32]. Taken together, these results suggest that more data is needed in these two species to be able to completely assess the conservation and divergence of their phosphoproteomes. Furthermore, the candidate StD sites might have specific regulatory properties that still have to be characterized and understood. A better understanding of these properties will allow us to make an important step towards in our attempt to describe and explain how small regulatory differences map to the important phenotypic differences among species. Mouse is the best model system to study human biology and diseases. It is therefore important to understand how these two species diverge and phosphoregulatory evolution may play an important role.

We identified sites that are phosphorylated in one species but that have diverged in the other so that the site is not phosphorylatable (SiD sites). While the biological meaning of the majority of these sites still remains to be assessed, our analysis suggests that many of them could be functionally redundant. This result supports the finding by Moses and collaborators that phosphorylation site evolutionary turnover has a role in shaping phosphoregulation [29]. If the redundancy hypothesis holds true, we might need to revisit estimations of phosphorylation conservation, since omitting positional redundancy may lead to an underestimation of phosphorylation site functional conservation. Moreover, this implies that we should consider different categories of phosphorylation sites: the ones for which the position along the protein is a determinant for their function (positionally-dependent phosphorylation sites) and those for which the global charge rather than the exact position is responsible for their function (positionally-flexible phosphorylation sites).

## Methods

### Phosphoproteomics and sequence data

An extensive dataset of human and mouse phosphorylation sites was built by combining data from 7 different databases and experimental studies [6], [32]–[37]. All protein sequences and orthology relationships were retrieved from ENSEMBL (version 69). In this study, only protein sequences for which we could find orthology relationships between a human protein and at least a mouse, dog and opossum protein were considered. This step allowed us to study the evolutionary history of phosphorylation sites. For humans and mouse, orthology relationships were determined for the longest isoforms of each protein. Each group of orthologous sequences was aligned using MUSCLE [57]. Disordered and ordered regions of proteins were predicted using DISOPRED [58]. In order to map phosphorylation sites to our sequences, the following procedure was applied. The sites that were already mapped onto proteins associated with ENSEMBL IDs in the original datasets were directly mapped to our sequences. For all other cases, phosphopeptides were mapped onto proteins using BLAT [59]. All peptides that mapped to more than one protein were removed at this step. Mapped phosphorylation sites and information about protein disorder are available in Dataset S2.

### Calculating random expectations for phosphorylation sites

In order to calculate the random expectation for the number of sites belonging to each one of the different categories (StC, StD and SiD), statuses (0: non-phosphorylated, 1: phosphorylated) of phosphorylatable amino acid were shuffled in each protein by preserving the overall proportion of sites for each residue (S, T or Y) and the localization in disordered/ordered regions. The null distributions were estimated by iterating this procedure 100 times, calculating each time the number of sites belonging to each category. We calculated random expectations by shuffling the mouse sites only. We also performed the calculations by independently shuffling both human and mouse sites and found similar results.

### Protein abundance data and classes of abundance

Data on protein abundance were taken from PaxDb [60] (*H. sapiens* whole organism integrated dataset). In the analysis presented on Figure 1D, proteins were ordered by their abundance and divided in four equal bins.

### Housekeeping proteins, tissue specific proteins and sites with known function

Data on housekeeping genes were retrieved from Eisenberg and Levanon [39] who identified 575 human genes that are expressed in 47 different tissues and cell lines based on microarray data. Data on tissue-specific genes derive from an independent dataset and were retrieved from the TiGER database [40]. About 5.3 millions human ESTs were mapped to UniGene clusters and the expression pattern of the all UniGenes in 30 human tissues was determined using the NCBI EST database. 7261 tissue-specific genes were identified. Manually curated data on functional phosphorylation sites (n = 156) were retrieved from Landry *et al.* [17]. These sites were derived from the manual curation of the primary literature.

### NetPhorest and position weight matrices scores

NetPhorest [41] was downloaded from (http://netphorest.info) and was run locally using default options. In order to calculate position weight matrices scores, 29 position weight matrices which scores are based on the same metric were obtained from Benjamin Turk [42]–[53]. These matrices were used to score all 10-mer amino acids in the mouse and human proteomes that have a phosphorylatable amino acid on the sixth position. The score reflects the probability of each 10-mer to be phosphorylated by a specific kinase.

### Comparison of proportions, distributions and correlations

Proportions were compared with 2-sample tests for equality of proportions with continuity correction. Distributions were compared with non-parametric Wilcoxon Rank Sum tests. Correlations were calculated with the Spearman method. All these statistical analyses were performed as implemented in R [61].

### Algorithm to identify evolutionary clustered sites phosphorylation sites pairs

Site colocalization in orthologous proteins was estimated using a window of positions (centered on each human phosphorylation site). The fraction of colocalized sites over the total number of sites was calculated for a range of window sizes. In order to determine which sites were closer in sequence linear space than expected by chance alone, the mouse phosphorylation sites were shuffled in each protein by preserving the overall proportion of sites for each residue (S, T or Y) and disordered/ordered regions, and the fraction of colocalized sites was calculated for each window length. One thousand iterations were performed in order to generate the null model. Also, we masked all the positions in which a phosphorylatable amino acid was present at a given position in both human and mouse. Evolutionary clustered sites were defined as sites that were more likely to be colocalized than expected by chance alone (null model). The closest pair of phosphorylation sites present in these windows was then selected (see also Figure S8). The phosphorylatable amino acids serine (S) and threonine (T) differ in biochemical properties compared to tyrosine (Y), another phosphorylatable amino acid [62]. Therefore, S/T and Y sites were considered as belonging to separate classes and not considered to be able to compensate each other. Only 1529 pairs of orthologous proteins that had at least two phosphorylation sites that diverged (site-divergence) in human and mouse respectively were considered. Among these pairs, 563 had at least one SiD site that involves a phospho-serine or phospho-threonine in both humans and mouse. Only one single pair had a SiD site that involves a phospho-tyrosine in both humans and mouse.

### Testing if evolutionary clustered sites tend to be phosphorylated by the same kinase or group of kinases

The kinase that was the most likely to phosphorylate each one of the evolutionary clustered sites was inferred using NetPhorest [41] and proportion of evolutionary clustered site pairs phosphorylated by the same kinase was determined. This number was compared to a null distribution obtained by randomly shuffling (10,000 iterations) the kinase-phosphorylation site associations between different evolutionary clustered sites. Analogous analyses were performed for StC and StD sites. We then performed the same analysis but this time using the three best kinases predicted by NetPhorest, as proposed by Tan *et al.* [19]. We therefore considered two evolutionary clustered sites as being phosphorylated by the same group of kinases if they shared one or more kinases (kinase group) among the three best kinases predicted to be associated to each site according to NetPhorest. This number was compared to a null distribution obtained by randomly shuffling (100 iterations) the kinases-phosphorylation site associations between different evolutionary clustered sites. Analogous analyses were performed for StC and StD sites. Finally, we performed again all the analyses described above but this time using position weight matrices from the literature (see section NetPhorest and position weight matrices scores for further details) instead of NetPhorest to infer the kinase that was the most likely to phosphorylate each one of the StD, StC and evolutionary clustered sites.

## Supporting Information

Dataset S1 Alignments of orthologous mammalian proteins for the 68 proteins that show significant clustering of SiD phosphorylation sites (i.e. that contain evolutionary clustered sites). Proteins' ENSEMBL IDs of the aligned proteins are provided. Alignments are in table format. The columns' IDs provide information about the organism (following the ENSEMBL convention; e.g. “hsa” indicates *Homo sapiens* and “mus”, *Mus musculus*) and the type of data included (“aa” for amino acid and “p” for phosphorylation).
(TXT)

Dataset S2 Human and mouse phosphorylation sites for the 11,150 proteins present in our dataset (i.e. that contains evolutionary clustered sites). ENSEMBL IDs of the aligned proteins are provided. The alignment is in table format. The column IDs provide information on the organism (following the ENSEMBL convention; e.g. “hsa” indicates *Homo sapiens* and “mus”, *Mus musculus*) and the type of data included (“aa” for amino acid and “p” for phosphorylation, “diso” for disorder/order). Two columns (“hsa” and “mus”) provide information about the position of the residues along the human or mouse sequences. Protein disorder is indicated by the “*” symbol, while order is indicated by the “.” symbol. For phosphorylation sites, we provide information (columns <organism_id>.p.db) about the papers/dataset that lists the site as being phosphorylated. (“Be”, Beltrao *et al.*, 2012; “Hp”, Keshava Prasad *et al.*, 2009; “Hu”, Huttlin *et al.*, 2010; “Mi”, Minguez *et al.*, 2012; “Pe”, Dinkel *et al.*, 2011; “Ph”, Gnad *et al.*, 2011; “Po”, Hornbeck *et al.*, 2012).
(TXT)

Figure S1 Comparison of human and mouse phosphorylation sites present in our dataset. (A) Global number of phosphorylation sites. (B) Proportion of the different phosphorylated residues (S: serine, T: threonine, Y: tyrosine).
(PDF)

Figure S2 Localization of SiD, StC and StD sites. Fraction of sites located in disordered, ordered or mixed regions for each of the three categories and comparison with the expectations. Mixed regions are regions where one site is located in a disordered region while the orthologous one is located in an ordered region.
(PDF)

Figure S3 Conservation and divergence of clusters of poly-S/T/Y. There are 158,970 poly-S/T/Y clusters (stretches of two or more consecutive S/T/Y residues) in the human proteome and 158,022 in the mouse. We defined three categories of clusters: i) Site-diverged clusters (SiD-c): human or mouse clusters that do not overlap with a cluster in the other species, even though they can overlap with single phosphorylation sites; ii) state-conserved clusters (StC-c): overlapping human and mouse clusters in which both the human and the mouse clusters contain at least one phosphorylation site: iii) state-diverged clusters (StD-c): overlapping human and mouse clusters in which only one among the human and the mouse clusters contains at least one phosphorylation site. The plots show the number of observed SiD-c, StC-c and StD-c clusters of poly-S/T/Y (orange dots) and the comparison to random expectations (distributions in grey). The null model was generated by 1,000 iterations in which human and mouse clusters were randomized.
(PDF)

Figure S4 Analysis of position weight matrice (PWM) scores for the different classes of sites and probability of being phosphorylated by the same protein kinase. (A) Comparison of the distributions of PWM scores for human and mouse phosphorylated and non-phosphorylated residues (Wilcoxon tests). (B) Comparison of the distributions of PWM scores for StD sites (Wilcoxon tests; *: p-value<0.05; **: p-value<0.01; ***: p-value<0.001). (C) Correlation between human and mouse PWM scores for StC sites (black) and StD sites phosphorylated in human but not in mouse (red). (D) Correlation between human and mouse PWM scores for StC sites (black) and StD sites phosphorylated in mouse but not in human (red). (E) Proportion of phosphorylated sites that have higher PWM scores compared to their corresponding site in the other species for StC and StD sites. (F) Proportion of sites phosphorylated by the same kinase for the different categories of sites (StD: state diverged, StC: state conserved, ECS: evolutionary clustered sites). Black dots represent the observed proportion. Orange lines represent the range of proportions expected by chance. The histogram shows the distribution of random expectations for ECS. P-value for StD and StC: <0.00001; p-value for ECS: 0.006.
(PDF)

Figure S5 NetPhorest scores and phosphorylation sites. Fraction of phosphorylated sites (human and mouse) as a function of the NetPhorest score.
(PDF)

Figure S6 Distributions of NetPhorest scores for the different classes of sites. (A,B) Distribution of NetPhorest scores for StC, StD and non-phosphorylated sites. Non-phosphorylated sites (red) are orthologous sites that are conserved at the residue level and both non-phosphorylated according to our phosphoproteomics data. For StD sites (in which one site is phosphorylated while the orthologous one is phosphorylatable but not phosphorylated) we present two distributions: one for phosphorylated residues and another for non-phosphorylated residues.
(PDF)

Figure S7 Relationship between NetPhorest scores in state-conserved sites and protein abundance. Distributions of NetPhorest scores for state-conserved sites (only the scores for the human residue were considered) for four classes of relative protein abundance.
(PDF)

Figure S8 Algorithm to detect evolutionary clustered sites (ECS). (A) Estimation of the colocalization of phosphorylation sites inside a window of length L. Calculations were performed for windows of amino acids of increasing length. (B) Shuffling of phosphorylation sites respecting their biochemical properties (residue: S, T or Y; location in ordered/disordered regions) and calculation of the null expectations for the colocalization inside a window of length L. Calculations were performed for windows of amino acids of increasing length. (C) Comparison of the observed and expected values of colocalization. (D) Determination of the closest phosphorylation sites for which the observed colocalization score is higher than expected by chance (null expectation).
(PDF)

Figure S9 Distribution of the number of evolutionary clustered sites per protein.
(PDF)

Figure S10 Distance between evolutionary clustered sites. (A) Proportion of evolutionary clustered sites as a function of the length of the window (expressed in number of amino acids) in which the clustered sites are contained. (B) Cumulative distribution of the proportion of evolutionary clustered sites as a function of the distance between them (1–100 aa).
(PDF)

Figure S11 Relationship between evolutionary clustered sites and available sites. (A) Proportion of protein pairs having evolutionary clustered sites as a function of the available sites (SiD sites). (B) Distribution of available sites present in the proteins that have evolutionary clustered sites.
(PDF)

Table S1 Number of phosphoproteins and phosphorylation sites (sorted by phosphorylatable residue) for all the studies we considered as well as the corresponding non-redundant values.
(DOCX)

Table S2 Comparison of StC and StD sites. The first ten site pairs present in the table are the pairs of StC sites with the highest NetPhorest scores. The last ten pairs are the pairs of StD sites with the highest difference of NetPhorest scores between the phosphorylated site and its non-phosphorylated counterpart. Green rows refer to phosphorylated sites while grey to non-phosphorylated ones. Differences between orthologous 15mers centered on each site are highlighted in yellow.
(DOCX)

Table S3 List of evolutionary clustered sites. The list includes for each pair of evolutionary clustered sites the name of the proteins, a description of the protein and the two identifiers.
(DOCX)

Table S4 List of proteins with more than two evolutionary clustered sites. The list includes for each pair of evolutionary clustered sites the name of the proteins where they are found, a description of the protein and the two identifiers of the sites.
(DOCX)
