# Supplementary figures and images for: Functional Divergence and Evolutionary Turnover in Mammalian Phosphoproteomes
Source: PLoS Genet. 2014 Jan 23;10(1):e1004062. doi: 10.1371/journal.pgen.1004062 (PMC3900387; doi:10.1371/journal.pgen.1004062)

**A**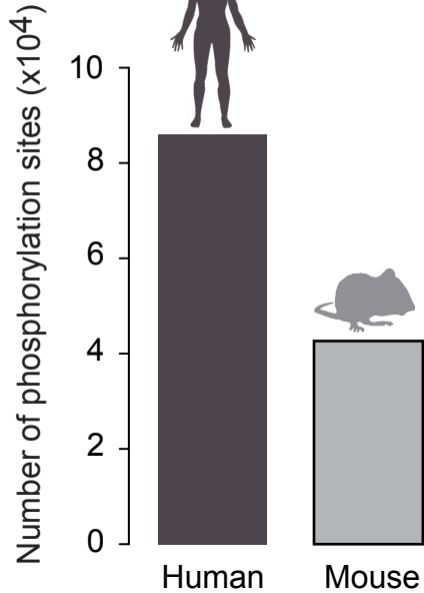**B**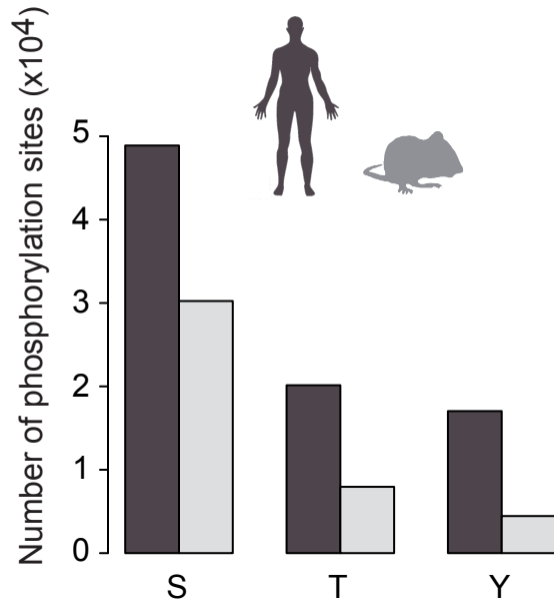

Supplement: Figure S1 — Comparison of human and mouse phosphorylation sites present in our dataset. (A) Global number of phosphorylation sites. (B) Proportion of the different phosphorylated residues (S: serine, T: threonine, Y: tyrosine). (PDF) [file pgen.1004062.s003.pdf]

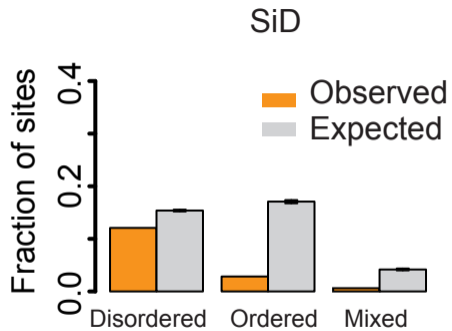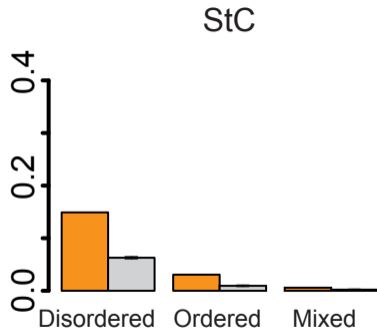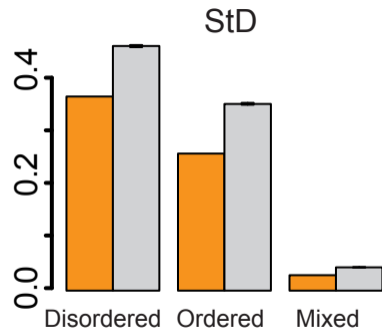

Supplement: Figure S2 — Localization of SiD, StC and StD sites. Fraction of sites located in disordered, ordered or mixed regions for each of the three categories and comparison with the expectations. Mixed regions are regions where one site is located in a disordered region while the orthologous one is located in an ordered region. (PDF) [file pgen.1004062.s004.pdf]

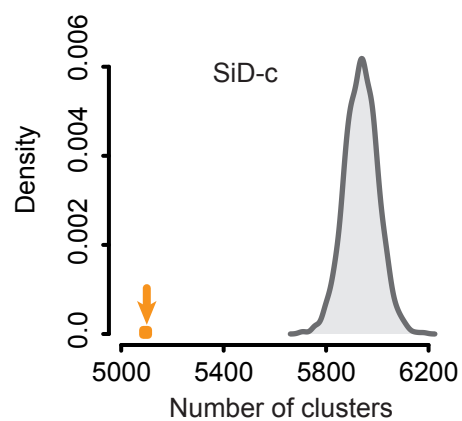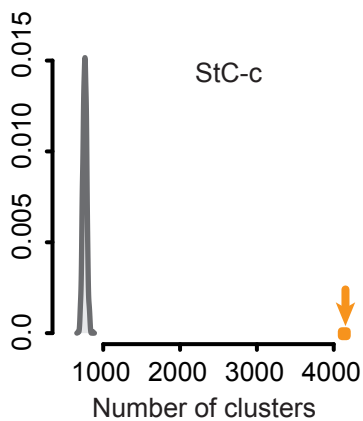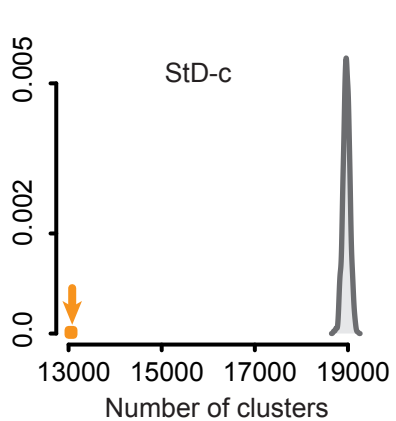

Supplement: Figure S3 — Conservation and divergence of clusters of poly-S/T/Y. There are 158,970 poly-S/T/Y clusters (stretches of two or more consecutive S/T/Y residues) in the human proteome and 158,022 in the mouse. We defined three categories of clusters: i) Site-diverged clusters (SiD-c): human or mouse clusters that do not overlap with a cluster in the other species, even though they can overlap with single phosphorylation sites; ii) state-conserved clusters (StC-c): overlapping human and mouse clusters in which both the human and the mouse clusters contain at least one phosphorylation site: iii) state-diverged clusters (StD-c): overlapping human and mouse clusters in which only one among the human and the mouse clusters contains at least one phosphorylation site. The plots show the number of observed SiD-c, StC-c and StD-c clusters of poly-S/T/Y (orange dots) and the comparison to random expectations (distributions in grey). The null model was generated by 1,000 iterations in which human and mouse clusters were randomized. (PDF) [file pgen.1004062.s005.pdf]

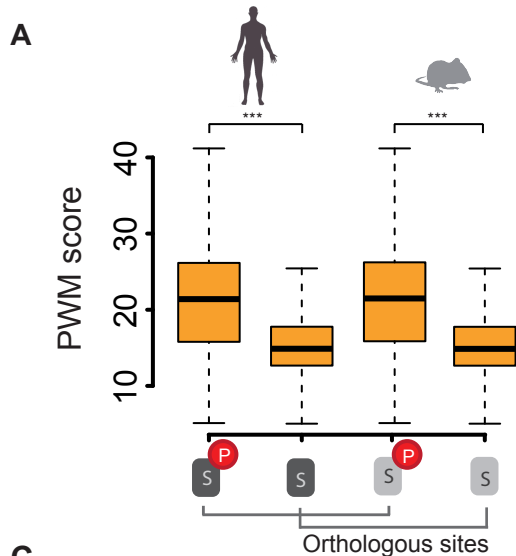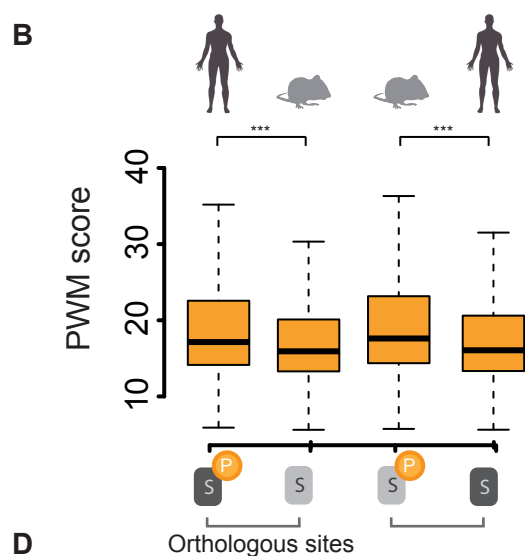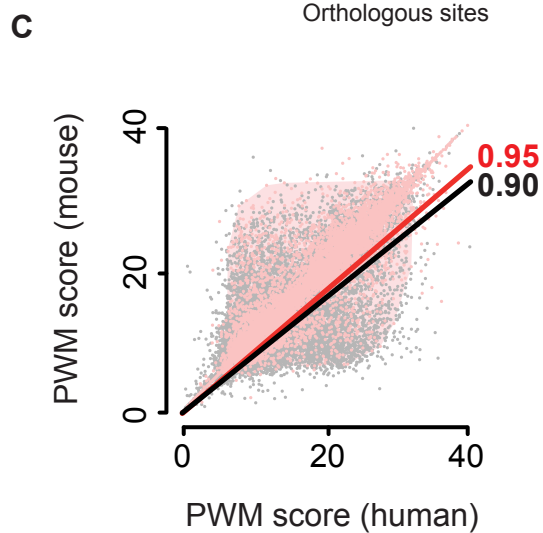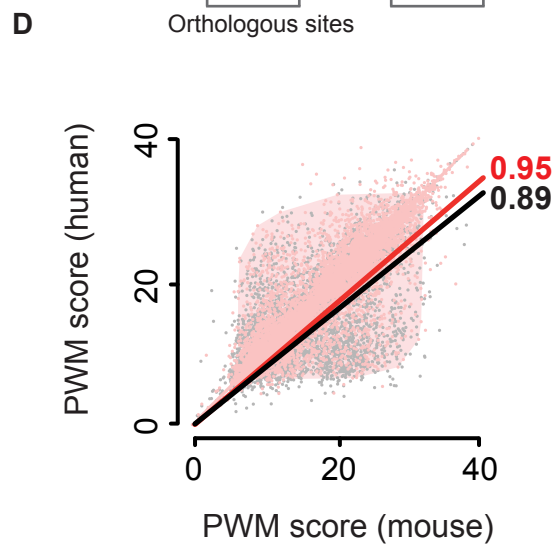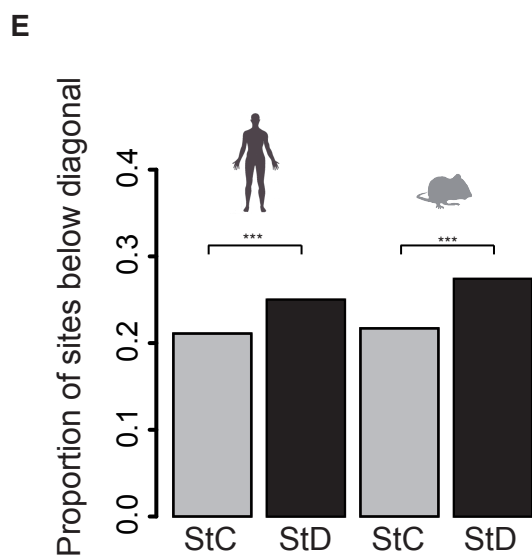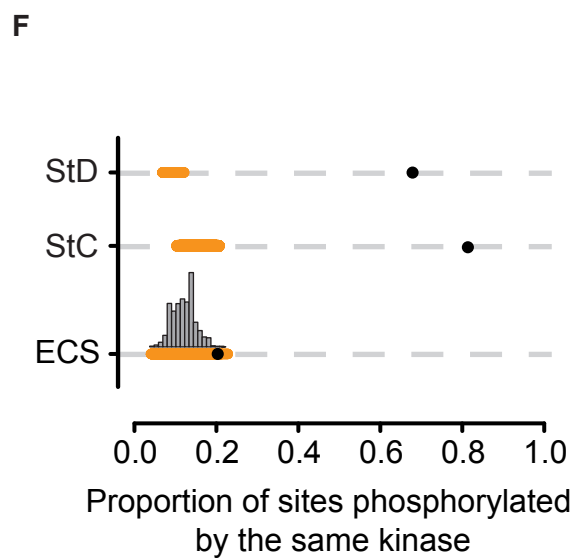

Supplement: Figure S4 — Analysis of position weight matrice (PWM) scores for the different classes of sites and probability of being phosphorylated by the same protein kinase. (A) Comparison of the distributions of PWM scores for human and mouse phosphorylated and non-phosphorylated residues (Wilcoxon tests). (B) Comparison of the distributions of PWM scores for StD sites (Wilcoxon tests; *: p-value<0.05; **: p-value<0.01; ***: p-value<0.001). (C) Correlation between human and mouse PWM scores for StC sites (black) and StD sites phosphorylated in human but not in mouse (red). (D) Correlation between human and mouse PWM scores for StC sites (black) and StD sites phosphorylated in mouse but not in human (red). (E) Proportion of phosphorylated sites that have higher PWM scores compared to their corresponding site in the other species for StC and StD sites. (F) Proportion of sites phosphorylated by the same kinase for the different categories of sites (StD: state diverged, StC: state conserved, ECS: evolutionary clustered sites). Black dots represent the observed proportion. Orange lines represent the range of proportions expected by chance. The histogram shows the distribution of random expectations for ECS. P-value for StD and StC: <0.00001; p-value for ECS: 0.006. (PDF) [file pgen.1004062.s006.pdf]

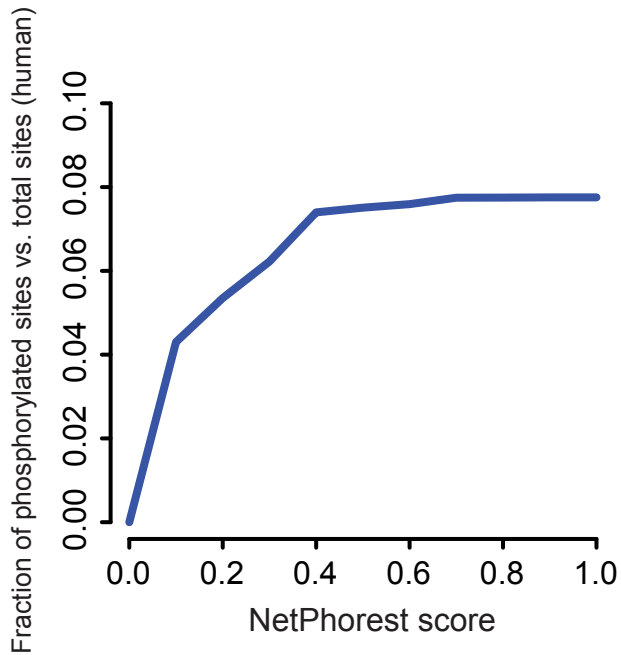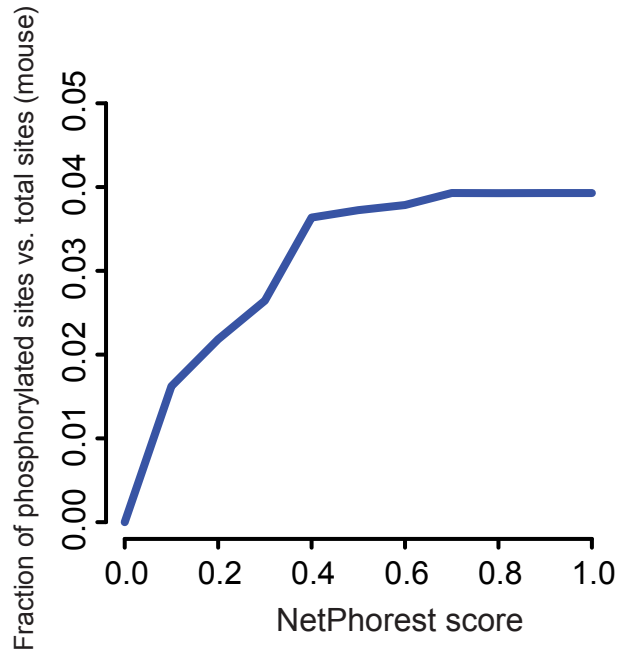

Supplement: Figure S5 — NetPhorest scores and phosphorylation sites. Fraction of phosphorylated sites (human and mouse) as a function of the NetPhorest score. (PDF) [file pgen.1004062.s007.pdf]

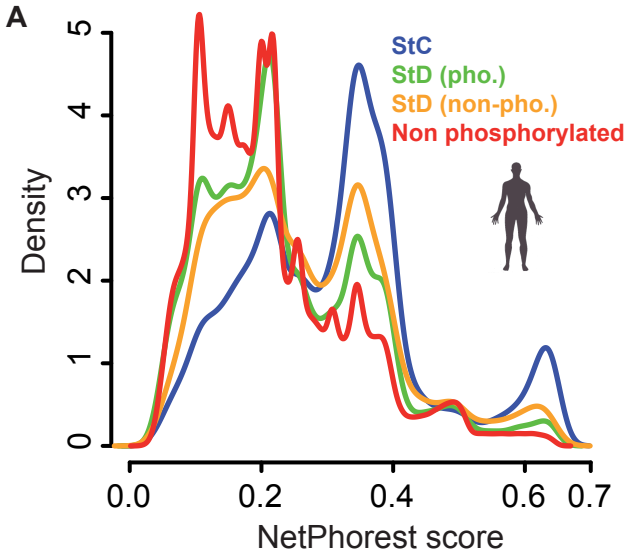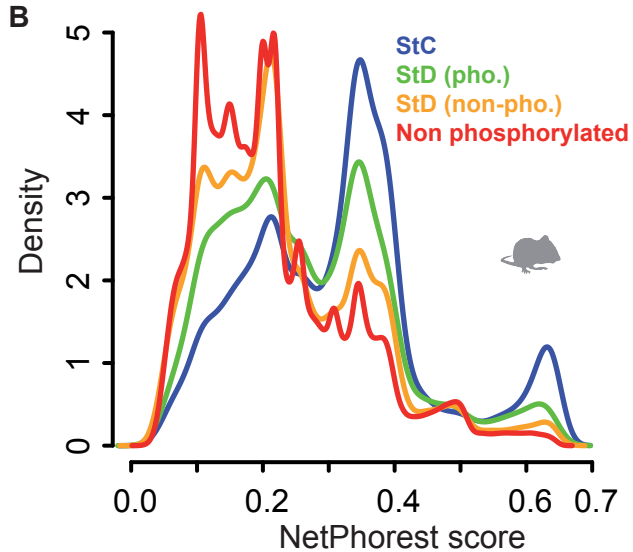

Supplement: Figure S6 — Distributions of NetPhorest scores for the different classes of sites. (A,B) Distribution of NetPhorest scores for StC, StD and non-phosphorylated sites. Non-phosphorylated sites (red) are orthologous sites that are conserved at the residue level and both non-phosphorylated according to our phosphoproteomics data. For StD sites (in which one site is phosphorylated while the orthologous one is phosphorylatable but not phosphorylated) we present two distributions: one for phosphorylated residues and another for non-phosphorylated residues. (PDF) [file pgen.1004062.s008.pdf]

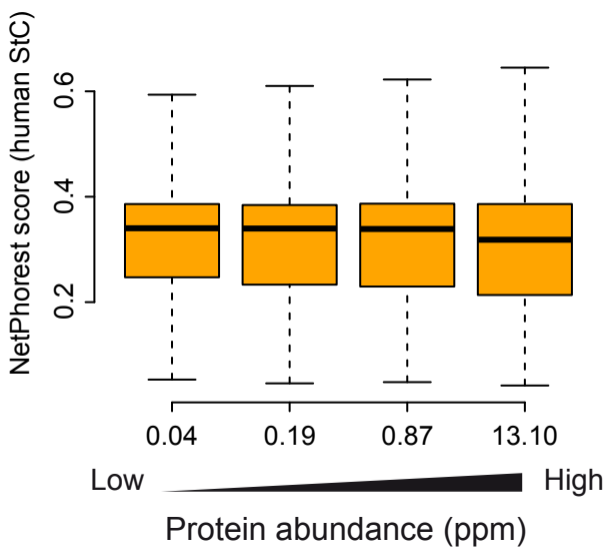

Supplement: Figure S7 — Relationship between NetPhorest scores in state-conserved sites and protein abundance. Distributions of NetPhorest scores for state-conserved sites (only the scores for the human residue were considered) for four classes of relative protein abundance. (PDF) [file pgen.1004062.s009.pdf]

**A**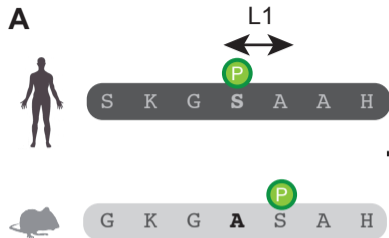

Colocalization

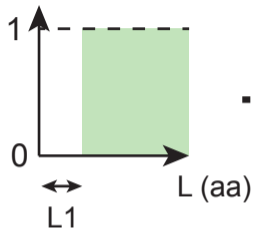**C**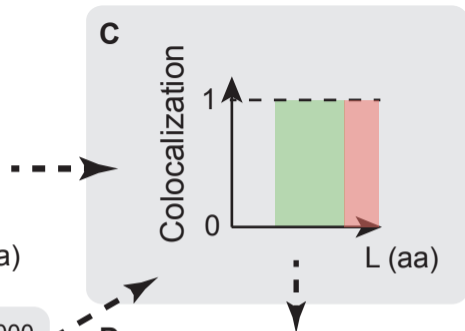**B**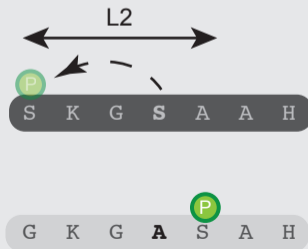

Colocalization

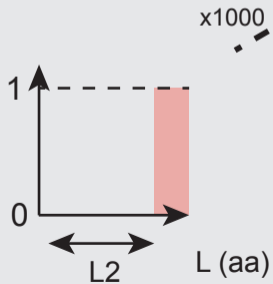

x1000

**D**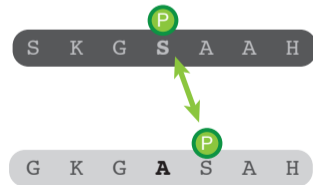

Supplement: Figure S8 — Algorithm to detect evolutionary clustered sites (ECS). (A) Estimation of the colocalization of phosphorylation sites inside a window of length L. Calculations were performed for windows of amino acids of increasing length. (B) Shuffling of phosphorylation sites respecting their biochemical properties (residue: S, T or Y; location in ordered/disordered regions) and calculation of the null expectations for the colocalization inside a window of length L. Calculations were performed for windows of amino acids of increasing length. (C) Comparison of the observed and expected values of colocalization. (D) Determination of the closest phosphorylation sites for which the observed colocalization score is higher than expected by chance (null expectation). (PDF) [file pgen.1004062.s010.pdf]

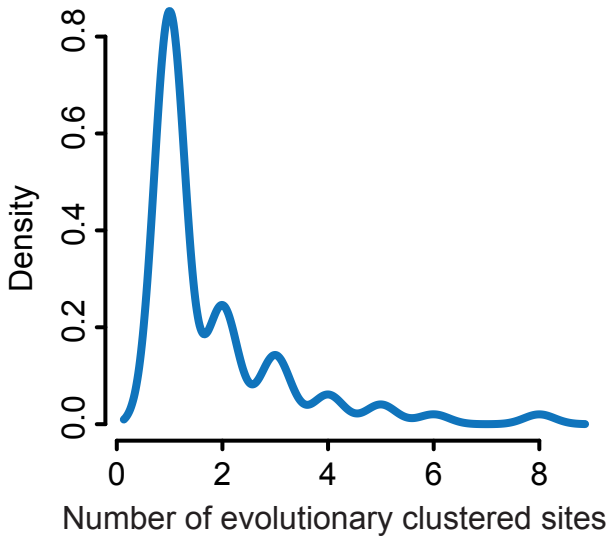

Supplement: Figure S9 — Distribution of the number of evolutionary clustered sites per protein. (PDF) [file pgen.1004062.s011.pdf]

**A**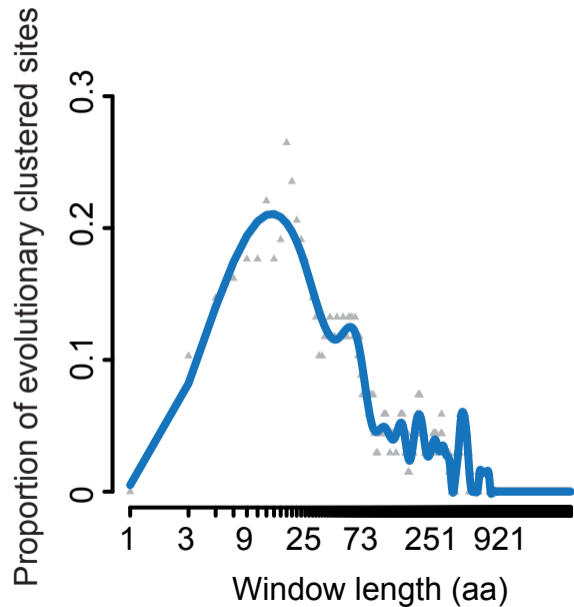**B**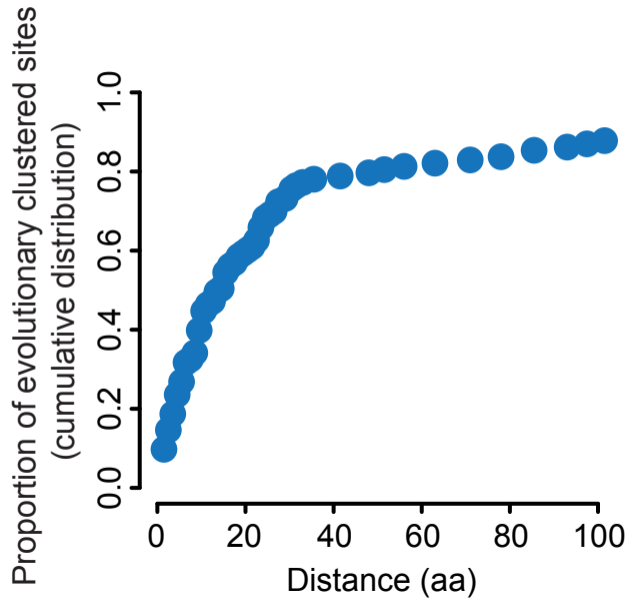

Supplement: Figure S10 — Distance between evolutionary clustered sites. (A) Proportion of evolutionary clustered sites as a function of the length of the window (expressed in number of amino acids) in which the clustered sites are contained. (B) Cumulative distribution of the proportion of evolutionary clustered sites as a function of the distance between them (1–100 aa). (PDF) [file pgen.1004062.s012.pdf]

**A**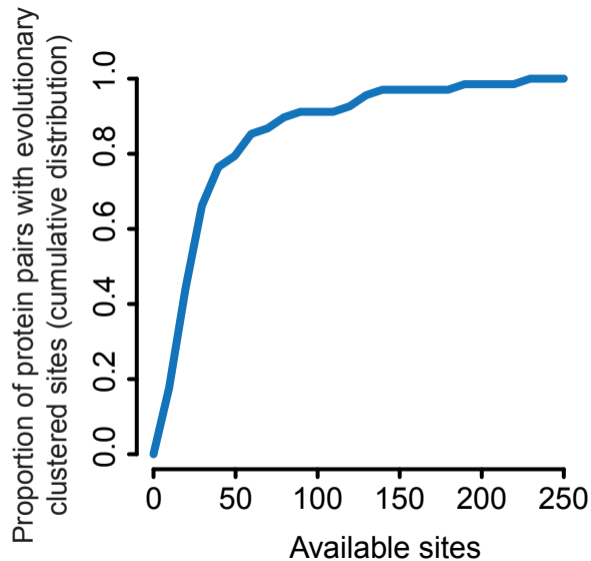**B**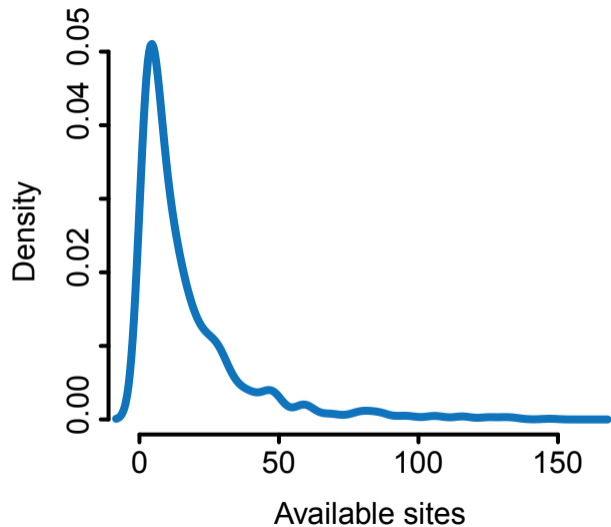

Supplement: Figure S11 — Relationship between evolutionary clustered sites and available sites. (A) Proportion of protein pairs having evolutionary clustered sites as a function of the available sites (SiD sites). (B) Distribution of available sites present in the proteins that have evolutionary clustered sites. (PDF) [file pgen.1004062.s013.pdf]
