# Supplementary material for: Functional Divergence and Evolutionary Turnover in Mammalian Phosphoproteomes
Source: PLoS Genet. 2014 Jan 23;10(1):e1004062. doi: 10.1371/journal.pgen.1004062 (PMC3900387; doi:10.1371/journal.pgen.1004062)
Supplement: Table S1 — Number of phosphoproteins and phosphorylation sites (sorted by phosphorylatable residue) for all the studies we considered as well as the corresponding non-redundant values. (DOCX) [file pgen.1004062.s014.docx]

|  | **Human** | | | | **Mouse** | | | |
| --- | --- | --- | --- | --- | --- | --- | --- | --- |
| **Study** | **#prot** | **S** | **T** | **Y** | **#prot** | **S** | **T** | **Y** |
| Minguez et al. | 5899 | 25799 | 8179 | 6631 | 3278 | 11474 | 2689 | 1708 |
| Beltrao et al. | 7349 | 28666 | 9240 | 8460 | 5760 | 21876 | 4706 | 2361 |
| Phosida et al. | 2312 | 7948 | 2056 | 454 | 2818 | 10001 | 1704 | 238 |
| HPRD | 4670 | 21493 | 6511 | 2340 | - | - | - | - |
| Phosphosite.Org | 8355 | 39796 | 16160 | 15610 | 5607 | 26010 | 6538 | 3958 |
| phosphoELM | 2923 | 11085 | 2743 | 1203 | 1510 | 3070 | 636 | 379 |
| Huttlin et al. | - | - | - | - | 3193 | 14679 | 2849 | 426 |
| Non-redundant | 12341 | 61401 | 24257 | 20962 | 8179 | 38718 | 9821 | 5501 |
