# Supplementary material for: Functional Divergence and Evolutionary Turnover in Mammalian Phosphoproteomes
Source: PLoS Genet. 2014 Jan 23;10(1):e1004062. doi: 10.1371/journal.pgen.1004062 (PMC3900387; doi:10.1371/journal.pgen.1004062)
Supplement: Table S2 — Comparison of StC and StD sites. The first ten site pairs present in the table are the pairs of StC sites with the highest NetPhorest scores. The last ten pairs are the pairs of StD sites with the highest difference of NetPhorest scores between the phosphorylated site and its non-phosphorylated counterpart. Green rows refer to phosphorylated sites while grey to non-phosphorylated ones. Differences between orthologous 15mers centered on each site are highlighted in yellow. (DOCX) [file pgen.1004062.s015.docx]

| **Protein** | **Site** | **Sequence** | **NetPhorest score** | **Predicted kinase** |
| --- | --- | --- | --- | --- |
| NUCL_HUMAN | ENSP00000318195_28 | PKEVEEDSEDEEMSE | 0.649539 | CK2 |
| NUCL_MOUSE | ENSMUSP00000027438_28 | PKEVEEDSEDEEMSE | 0.649539 | CK2 |
|  |  |  |  |  |
| PAXB1_HUMAN | ENSP00000328992_262 | REDENDASDDEDDDE | 0.649397 | CK2 |
| PAXB1_MOUSE | ENSMUSP00000113835_264 | REDENDASDDEDDDE | 0.649397 | CK2 |
|  |  |  |  |  |
| ARI4A_HUMAN | ENSP00000347602_160 | DEKEEESSEEEDEDK | 0.649337 | CK2 |
| ARI4A_MOUSE | ENSMUSP00000035512_160 | DEKEEESSEEEDEDK | 0.649337 | CK2 |
|  |  |  |  |  |
| B3KYA7_HUMAN | ENSP00000429744_344 | LEEEEENSDEDELDS | 0.648665 | CK2 |
| B3KYA7_MOUSE | ENSMUSP00000018476_316 | LEEEEENSDEDELDS | 0.648665 | CK2 |
|  |  |  |  |  |
| RPC7L_HUMAN | ENSP00000358320_163 | KKEEEVTSEEDEEKE | 0.648586 | CK2 |
| RPC7L_MOUSE | ENSMUSP00000089544_163 | KKEEEVTSEEDEEKE | 0.648586 | CK2 |
|  |  |  |  |  |
| ARI4A_HUMAN | ENSP00000347602_159 | EDEKEEESSEEEDED | 0.648118 | CK2 |
| ARI4A_MOUSE | ENSMUSP00000035512_159 | EDEKEEESSEEEDED | 0.648118 | CK2 |
|  |  |  |  |  |
| ARI4B_HUMAN | ENSP00000355562_295 | EKEKEDNSSEEEEEI | 0.647742 | CK2 |
| ARI4B_MOUSE | ENSMUSP00000106163_295 | EKEKEDNSSEEEEEI | 0.647742 | CK2 |
|  |  |  |  |  |
| SENP3_HUMAN | ENSP00000403712_75 | PSFDASASEEEEEEE | 0.647679 | CK2 |
| SENP3_MOUSE | ENSMUSP00000005336_73 | PSFDASASEEEEEEE | 0.647679 | CK2 |
|  |  |  |  |  |
| TBD2B_HUMAN | ENSP00000300584_957 | PDKGELVSDEEEDT | 0.647633 | CK2 |
| TBD2B_MOUSE | ENSMUSP00000045413_959 | PDKGELVSDEEEDT | 0.647633 | CK2 |
|  |  |  |  |  |
| U5S1_HUMAN | ENSP00000392094_19 | YIGPELDSDEDDDEL | 0.647600 | CK2 |
| U5S1_MOUSE | ENSMUSP00000021306_19 | YIGPELDSDEDDDEL | 0.647600 | CK2 |
|  |  |  |  |  |
| NIN_HUMAN | ENSP00000245441_1145 | VTRRHVLSDLEDDEV | 0.632661 | CK2 |
| NIN_MOUSE | ENSMUSP00000082422_1133 | PATKHFLSDLGDHEA | 0.103702 | DMPK |
|  |  |  |  |  |
| F111A_HUMAN | ENSP00000434435_607 | QQDVEMMSDEDL | 0.633808 | CK2 |
| F111A_MOUSE | ENSMUSP00000119518_610 | VQNVEMLSIDF | 0.139290 | CK2 |
|  |  |  |  |  |
| OSTP_HUMAN | ENSP00000378517_191 | ATDEDITSHMESEEL | 0.601545 | CK2 |
| OSTP_MOUSE | ENSMUSP00000031243_176 | ATDEDLTSHMKSGES | 0.107948 | CK1 |
|  |  |  |  |  |
| ORC2_HUMAN | ENSP00000234296_177 | LIVPRSHSDSESEYS | 0.576608 | CK2 |
| ORC2_MOUSE | ENSMUSP00000027198_176 | IIASRSHYDSESEYS | 0.090376 | MAP2K6_MAP2K3_MAP2K4_MAP2K7 |
|  |  |  |  |  |
| K1551_HUMAN | ENSP00000310338_1198 | NSIKNSSSEEEKQKE | 0.602660 | CK2 |
| K1551_MOUSE | ENSMUSP00000041180_956 | VPQCHCSSTEKKEKD | 0.119368 | ACTR2_ACTR2B_TGFbR2 |
|  |  |  |  |  |
| LTV1_HUMAN | ENSP00000356548_211 | YDSAGLLSDEDCMSV | 0.612627 | CK2 |
| LTV1_MOUSE | ENSMUSP00000019950_206 | RSSAGFLSDGGDLSA | 0.129578 | CK2 |
|  |  |  |  |  |
| RBP2_HUMAN | ENSP00000283195_2583 | KCELSKNSDIEQSSD | 0.593321 | CK2 |
| RBP2_MOUSE | ENSMUSP00000003310_2421 | KCELPQNSDIKQSSD | 0.115794 | GRK |
|  |  |  |  |  |
| SYCC_HUMAN | ENSP00000369897_264 | LTGEEVNSCVEVLLE | 0.567883 | CK2 |
| SYCC_MOUSE | ENSMUSP00000010899_264 | LSGEEVDSKVQVLL | 0.093388 | CK2 |
|  |  |  |  |  |
| TSN1_HUMAN | ENSP00000361072_156 | CCGFTNYTDFEDSPY | 0.585526 | CK2 |
| TSN1_MOUSE | ENSMUSP00000030465_156 | CCGFNNYTDFNASRF | 0.115104 | CK2 |
|  |  |  |  |  |
| SETB1_HUMAN | ENSP00000271640_474 | LSPQAGDSDLESQLA | 0.543830 | CK2 |
| SETB1_MOUSE | ENSMUSP00000015841_473 | LSPQAADTESLESQL | 0.080428 | CK2 |
