# Supplementary material for: Functional Divergence and Evolutionary Turnover in Mammalian Phosphoproteomes
Source: PLoS Genet. 2014 Jan 23;10(1):e1004062. doi: 10.1371/journal.pgen.1004062 (PMC3900387; doi:10.1371/journal.pgen.1004062)
Supplement: Table S3 — List of evolutionary clustered sites. The list includes for each pair of evolutionary clustered sites the name of the proteins, a description of the protein and the two identifiers. (DOCX) [file pgen.1004062.s016.docx]

| **Protein ID** | **Description** | **Human site** | **Mouse site** |
| --- | --- | --- | --- |
| PKP2 | plakophilin 2 | ENSP00000070846_203 | ENSMUSP00000036890_181 |
| PKP2 | plakophilin 2 | ENSP00000070846_267 | ENSMUSP00000036890_235 |
| PNN | pinin, desmosome associated protein | ENSP00000216832_552 | ENSMUSP00000021381_559 |
| NUP210 | nucleoporin 210kDa | ENSP00000254508_1862 | ENSMUSP00000032179_1839 |
| NUP210 | nucleoporin 210kDa | ENSP00000254508_1863 | ENSMUSP00000032179_1839 |
| VPS13C | vacuolar protein sorting 13 homolog C (S. cerevisiae) | ENSP00000261517_542 | ENSMUSP00000077040_839 |
| VPS13C | vacuolar protein sorting 13 homolog C (S. cerevisiae) | ENSP00000261517_734 | ENSMUSP00000077040_839 |
| VPS13C | vacuolar protein sorting 13 homolog C (S. cerevisiae) | ENSP00000261517_736 | ENSMUSP00000077040_839 |
| VPS13C | vacuolar protein sorting 13 homolog C (S. cerevisiae) | ENSP00000261517_1902 | ENSMUSP00000077040_1956 |
| DSG2 | desmoglein 2 | ENSP00000261590_984 | ENSMUSP00000057096_921 |
| ADNP2 | ADNP homeobox 2 | ENSP00000262198_1024 | ENSMUSP00000068560_1052 |
| LRP2 | low density lipoprotein receptor-related protein 2 | ENSP00000263816_4527 | ENSMUSP00000079752_4533 |
| LRP2 | low density lipoprotein receptor-related protein 2 | ENSP00000263816_4634 | ENSMUSP00000079752_4632 |
| EHBP1 | EH domain binding protein 1 | ENSP00000263991_751 | ENSMUSP00000105191_765 |
| EHBP1 | EH domain binding protein 1 | ENSP00000263991_769 | ENSMUSP00000105191_765 |
| ALMS1 | Alstrom syndrome 1 | ENSP00000264448_2751 | ENSMUSP00000071904_1916 |
| ALMS1 | Alstrom syndrome 1 | ENSP00000264448_2754 | ENSMUSP00000071904_1916 |
| NBN | nibrin | ENSP00000265433_402 | ENSMUSP00000029879_429 |
| NBN | nibrin | ENSP00000265433_497 | ENSMUSP00000029879_533 |
| NBN | nibrin | ENSP00000265433_516 | ENSMUSP00000029879_543 |
| FANCM | Fanconi anemia, complementation group M | ENSP00000267430_1413 | ENSMUSP00000054797_1379 |
| FANCM | Fanconi anemia, complementation group M | ENSP00000267430_1673 | ENSMUSP00000054797_1638 |
| FANCM | Fanconi anemia, complementation group M | ENSP00000267430_1686 | ENSMUSP00000054797_1638 |
| FANCM | Fanconi anemia, complementation group M | ENSP00000267430_1693 | ENSMUSP00000054797_1638 |
| FANCM | Fanconi anemia, complementation group M | ENSP00000267430_1721 | ENSMUSP00000054797_1638 |
| C10orf47 | chromosome 10 open reading frame 47 | ENSP00000277570_146 | ENSMUSP00000060780_225 |
| UHRF1BP1L | UHRF1 binding protein 1-like | ENSP00000279907_446 | ENSMUSP00000020112_797 |
| PDE3B | phosphodiesterase 3B, cGMP-inhibited | ENSP00000282096_561 | ENSMUSP00000032909_536 |
| RANBP2 | RAN binding protein 2 | ENSP00000283195_1146 | ENSMUSP00000003310_1141 |
| RANBP2 | RAN binding protein 2 | ENSP00000283195_2802 | ENSMUSP00000003310_2638 |
| RANBP2 | RAN binding protein 2 9848] | ENSP00000283195_2807 | ENSMUSP00000003310_2641 |
| ZNF646 | zinc finger protein 646 29004] | ENSP00000300850_1448 | ENSMUSP00000052641_1412 |
| CLSPN | claspin | ENSP00000312995_69 | ENSMUSP00000045344_84 |
| CLSPN | claspin | ENSP00000312995_949 | ENSMUSP00000045344_948 |
| CLSPN | claspin | ENSP00000312995_955 | ENSMUSP00000045344_948 |
| CLSPN | claspin | ENSP00000312995_1161 | ENSMUSP00000045344_1123 |
| DAB2 | disabled homolog 2, mitogen-responsive phosphoprotein (Drosophila) | ENSP00000313391_723 | ENSMUSP00000079689_731 |
| FAM123C | family with sequence similarity 123C | ENSP00000314914_307 | ENSMUSP00000054748_267 |
| MAP1S | microtubule-associated protein 1S | ENSP00000325313_582 | ENSMUSP00000019405_532 |
| MAP1S | microtubule-associated protein 1S | ENSP00000325313_640 | ENSMUSP00000019405_573 |
| MAP1S | microtubule-associated protein 1S | ENSP00000325313_643 | ENSMUSP00000019405_573 |
| DDX24 | DEAD (Asp-Glu-Ala-Asp) box polypeptide 24 | ENSP00000328690_302 | ENSMUSP00000105628_329 |
| LRRC16A | leucine rich repeat containing 16A | ENSP00000331983_1314 | ENSMUSP00000072662_1320 |
| EFCAB13 | EF-hand calcium binding domain 13 | ENSP00000332111_385 | ENSMUSP00000116040_452 |
| BMP2K | BMP2 inducible kinase | ENSP00000334836_728 | ENSMUSP00000037970_715 |
| BMP2K | BMP2 inducible kinase | ENSP00000334836_1011 | ENSMUSP00000037970_888 |
| BMP2K | BMP2 inducible kinase | ENSP00000334836_1080 | ENSMUSP00000037970_888 |
| KIF18B | kinesin family member 18B | ENSP00000341466_676 | ENSMUSP00000021311_558 |
| FGD6 | FYVE, RhoGEF and PH domain containing 6 | ENSP00000344446_553 | ENSMUSP00000020208_557 |
| FGD6 | FYVE, RhoGEF and PH domain containing 6 | ENSP00000344446_632 | ENSMUSP00000020208_557 |
| FGD6 | FYVE, RhoGEF and PH domain containing 6 | ENSP00000344446_693 | ENSMUSP00000020208_557 |
| HTT | huntingtin | ENSP00000347184_411 | ENSMUSP00000078945_638 |
| MKL1 | megakaryoblastic leukemia (translocation) 1 | ENSP00000347847_295 | ENSMUSP00000105207_335 |
| MKL1 | megakaryoblastic leukemia (translocation) 1 | ENSP00000347847_305 | ENSMUSP00000105207_345 |
| SVIL | supervillin | ENSP00000348128_221 | ENSMUSP00000115078_218 |
| SVIL | supervillin | ENSP00000348128_226 | ENSMUSP00000115078_218 |
| SVIL | supervillin | ENSP00000348128_240 | ENSMUSP00000115078_218 |
| SVIL | supervillin | ENSP00000348128_253 | ENSMUSP00000115078_218 |
| SVIL | supervillin | ENSP00000348128_253 | ENSMUSP00000115078_248 |
| SVIL | supervillin | ENSP00000348128_261 | ENSMUSP00000115078_248 |
| SVIL | supervillin | ENSP00000348128_263 | ENSMUSP00000115078_248 |
| SVIL | supervillin | ENSP00000348128_914 | ENSMUSP00000115078_857 |
| CLCC1 | chloride channel CLIC-like 1 | ENSP00000349456_506 | ENSMUSP00000102224_502 |
| CLCC1 | chloride channel CLIC-like 1 | ENSP00000349456_509 | ENSMUSP00000102224_503 |
| PDE3A | phosphodiesterase 3A, cGMP-inhibited | ENSP00000351957_475 | ENSMUSP00000038749_472 |
| PDE3A | phosphodiesterase 3A, cGMP-inhibited | ENSP00000351957_523 | ENSMUSP00000038749_526 |
| PCNT | pericentrin | ENSP00000352572_1703 | ENSMUSP00000001179_1444 |
| PCNT | pericentrin | ENSP00000352572_2370 | ENSMUSP00000001179_1990 |
| PARP9 | poly (ADP-ribose) polymerase family, member 9 | ENSP00000353512_61 | ENSMUSP00000110528_20 |
| C15orf39 | chromosome 15 open reading frame 39 | ENSP00000353854_586 | ENSMUSP00000034846_579 |
| RP5-862P8.2 | Mitogen-activated protein kinase kinase kinase MLK4 | ENSP00000355583_542 | ENSMUSP00000034316_521 |
| RP5-862P8.2 | Mitogen-activated protein kinase kinase kinase MLK4 | ENSP00000355583_546 | ENSMUSP00000034316_521 |
| PTPRC | protein tyrosine phosphatase, receptor type, C | ENSP00000356346_1281 | ENSMUSP00000027645_1265 |
| PTPRC | protein tyrosine phosphatase, receptor type, C | ENSP00000356346_1287 | ENSMUSP00000027645_1271 |
| CEP350 | centrosomal protein 350kDa | ENSP00000356579_1195 | ENSMUSP00000120085_1200 |
| CEP350 | centrosomal protein 350kDa | ENSP00000356579_1219 | ENSMUSP00000120085_1200 |
| CEP350 | centrosomal protein 350kDa | ENSP00000356579_2204 | ENSMUSP00000120085_2219 |
| CEP350 | centrosomal protein 350kDa | ENSP00000356579_2238 | ENSMUSP00000120085_2219 |
| CEP350 | centrosomal protein 350kDa | ENSP00000356579_2239 | ENSMUSP00000120085_2221 |
| F5 | coagulation factor V (proaccelerin, labile factor) | ENSP00000356770_1155 | ENSMUSP00000083204_903 |
| KNDC1 | kinase non-catalytic C-lobe domain (KIND) containing 1 | ENSP00000357561_257 | ENSMUSP00000050586_267 |
| SLK | STE20-like kinase | ENSP00000358770_569 | ENSMUSP00000049977_554 |
| DST | dystonin | ENSP00000359790_2635 | ENSMUSP00000110756_2521 |
| ZNF217 | zinc finger protein 217 | ENSP00000360526_568 | ENSMUSP00000104783_621 |
| ATRX | alpha thalassemia/mental retardation syndrome X-linked | ENSP00000362441_33 | ENSMUSP00000109203_43 |
| ATRX | alpha thalassemia/mental retardation syndrome X-linked | ENSP00000362441_52 | ENSMUSP00000109203_43 |
| ATRX | alpha thalassemia/mental retardation syndrome X-linked | ENSP00000362441_65 | ENSMUSP00000109203_35 |
| ATRX | alpha thalassemia/mental retardation syndrome X-linked | ENSP00000362441_706 | ENSMUSP00000109203_669 |
| ATRX | alpha thalassemia/mental retardation syndrome X-linked | ENSP00000362441_899 | ENSMUSP00000109203_871 |
| ATRX | alpha thalassemia/mental retardation syndrome X-linked | ENSP00000362441_1068 | ENSMUSP00000109203_1075 |
| ITPR3 | inositol 1,4,5-trisphosphate receptor, type 3 | ENSP00000363435_1861 | ENSMUSP00000038150_1831 |
| SPEN | spen homolog, transcriptional regulator (Drosophila) | ENSP00000364912_1622 | ENSMUSP00000101412_1669 |
| SPEN | spen homolog, transcriptional regulator (Drosophila) | ENSP00000364912_2014 | ENSMUSP00000101412_2107 |
| SPEN | spen homolog, transcriptional regulator (Drosophila) | ENSP00000364912_2486 | ENSMUSP00000101412_2402 |
| ATXN2 | ataxin 2 | ENSP00000366843_872 | ENSMUSP00000056715_830 |
| RTN3 | reticulon 3 | ENSP00000367050_268 | ENSMUSP00000065810_225 |
| HIVEP1 | human immunodeficiency virus type I enhancer binding protein 1 | ENSP00000368698_479 | ENSMUSP00000056147_591 |
| BRCA2 | breast cancer 2, early onset | ENSP00000369497_384 | ENSMUSP00000038576_400 |
| SHROOM2 | shroom family member 2 | ENSP00000370299_229 | ENSMUSP00000098701_237 |
| FANCA | Fanconi anemia, complementation group A | ENSP00000373952_850 | ENSMUSP00000045217_1071 |
| CCDC88C | coiled-coil domain containing 88C | ENSP00000374507_1023 | ENSMUSP00000082177_794 |
| ACD | adrenocortical dysplasia homolog (mouse) | ENSP00000377496_411 | ENSMUSP00000048180_290 |
| MYLK3 | myosin light chain kinase 3 | ENSP00000378288_450 | ENSMUSP00000034133_432 |
| GLI3 | GLI family zinc finger 3 | ENSP00000379258_850 | ENSMUSP00000106137_851 |
| CD44 | CD44 molecule (Indian blood group) | ENSP00000398632_686 | ENSMUSP00000005218_728 |
| CD44 | CD44 molecule (Indian blood group) | ENSP00000398632_717 | ENSMUSP00000005218_728 |
| CD44 | CD44 molecule (Indian blood group) | ENSP00000398632_717 | ENSMUSP00000005218_773 |
| GORASP2 | golgi reassembly stacking protein 2, 55kDa | ENSP00000410208_448 | ENSMUSP00000028509_432 |
| TOP2A | topoisomerase (DNA) II alpha 170kDa | ENSP00000411532_1360 | ENSMUSP00000068896_1379 |
| TOP2A | topoisomerase (DNA) II alpha 170kDa | ENSP00000411532_1361 | ENSMUSP00000068896_1379 |
| TOP2A | topoisomerase (DNA) II alpha 170kDa | ENSP00000411532_1392 | ENSMUSP00000068896_1367 |
| TOP2A | topoisomerase (DNA) II alpha 170kDa | ENSP00000411532_1495 | ENSMUSP00000068896_1469 |
| C9orf172 | chromosome 9 open reading frame 172 | ENSP00000412388_104 | ENSMUSP00000109855_105 |
| CAMSAP3 | calmodulin regulated spectrin-associated protein family, member 3 | ENSP00000416797_704 | ENSMUSP00000125993_583 |
| CAMSAP3 | calmodulin regulated spectrin-associated protein family, member 3 | ENSP00000416797_811 | ENSMUSP00000125993_882 |
| CCDC110 | coiled-coil domain containing 110 | ENSP00000427246_807 | ENSMUSP00000092964_644 |
| PRAGMIN | Tyrosine-protein kinase SgK223 | ENSP00000428054_148 | ENSMUSP00000106118_131 |
| RNF214 | ring finger protein 214 | ENSP00000431643_56 | ENSMUSP00000060941_48 |
| DMXL1 | Dmx-like 1 | ENSP00000439479_1841 | ENSMUSP00000045559_1829 |
| SLC1A5 | solute carrier family 1 (neutral amino acid transporter), member 5 | ENSP00000444408_9 | ENSMUSP00000104136_33 |
| C14orf38 | chromosome 14 open reading frame 38 | ENSP00000452964_279 | ENSMUSP00000021494_313 |
| OBSCN | obscurin, cytoskeletal calmodulin and titin-interacting RhoGEF | ENSP00000455507_3159 | ENSMUSP00000038264_3281 |
| MKL2 | MKL/myocardin-like 2 | ENSP00000459626_852 | ENSMUSP00000009713_846 |
