# Supplementary material for: Functional Divergence and Evolutionary Turnover in Mammalian Phosphoproteomes
Source: PLoS Genet. 2014 Jan 23;10(1):e1004062. doi: 10.1371/journal.pgen.1004062 (PMC3900387; doi:10.1371/journal.pgen.1004062)
Supplement: Table S4 — List of proteins with more than two evolutionary clustered sites. The list includes for each pair of evolutionary clustered sites the name of the proteins where they are found, a description of the protein and the two identifiers of the sites. (DOCX) [file pgen.1004062.s017.docx]

| **Protein ID** | **Description** | **Num. ECS** |
| --- | --- | --- |
| SVIL | supervillin | 8 |
| ATRX | alpha thalassemia/mental retardation syndrome X-linked | 6 |
| FANCM | Fanconi anemia, complementation group M | 5 |
| CEP350 | centrosomal protein 350kDa | 5 |
| TOP2A | topoisomerase (DNA) II alpha 170kDa | 4 |
| VPS13C | vacuolar protein sorting 13 homolog C (S. cerevisiae) | 4 |
| CLSPN | claspin | 4 |
| SPEN | spen homolog, transcriptional regulator (Drosophila) | 3 |
| BMP2K | BMP2 inducible kinase | 3 |
| NBN | nibrin | 3 |
| CD44 | CD44 molecule (Indian blood group) | 3 |
| MAP1S | microtubule-associated protein 1S | 3 |
| FGD6 | FYVE, RhoGEF and PH domain containing 6 | 3 |
| RBP2 | RAN binding protein 2 | 3 |
